# Broadening the Scope of Sapofection: Cationic Peptide-Saponin Conjugates Improve Gene Delivery *In Vitro* and *In Vivo*

**DOI:** 10.1021/acsami.4c05846

**Published:** 2024-07-06

**Authors:** Meike Kolster, Alexander Sonntag, Christoph Weise, Juan Correa, Hendrik Fuchs, Wolfgang Walther, Eduardo Fernandez-Megia, Alexander Weng

**Affiliations:** † Institut für Pharmazie, Freie Universität Berlin, Königin-Luise-Straße 2-4, Berlin 14195, Germany; ‡ Institut für Chemie und Biochemie, Freie Universität Berlin, Thielallee 63, Berlin 14195, Germany; § Centro Singular de Investigación en Química Biolóxica e Materiais Moleculares (CIQUS), Departamento de Química Orgánica, Universidade de Santiago de Compostela, Jenaro de la Fuente s/n, Santiago de Compostela 15782, Spain; ∥ Institut für Laboratoriumsmedizin, Klinische Chemie und Pathobiochemie, Charité − Universitätsmedizin Berlin, corporate member of Freie Universität Berlin and Humboldt-Universität zu Berlin, Augustenburger Platz 1, Berlin 13353, Germany; ⊥ Experimental Pharmacology & Oncology Berlin-Buch GmbH, Robert-Rössle-Str. 10, Berlin 13125, Germany

**Keywords:** gene therapy, endosomal escape, saponin, polylysine, SO1861

## Abstract

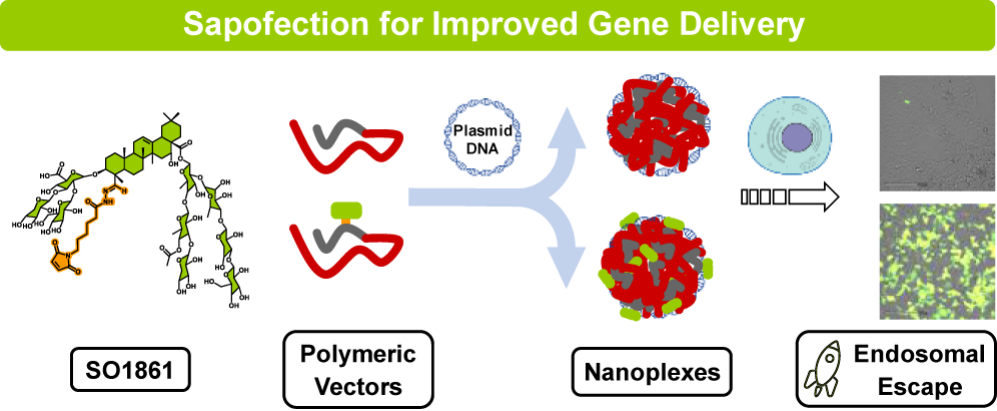

Gene therapies represent promising new therapeutic options for a variety of indications. However, despite several approved drugs, its potential remains untapped. For polymeric gene delivery, endosomal escape represents a bottleneck. SO1861, a naturally occurring triterpene saponin with endosomal escape properties isolated from *Saponaria officinalis* L., has been described as additive agent to enhance transfection efficiency (sapofection). However, the challenge to synchronize the saponin and gene delivery system *in vivo* imposes limitations. Herein, we address this issue by conjugating SO1861 to a peptide-based gene vector using a pH-sensitive hydrazone linker programmed to release SO1861 at the acidic pH of the endosome. Nanoplexes formulated with SO1861-equipped peptides were investigated for transfection efficiency and tolerability *in vitro* and *in vivo*. In all investigated cell lines, SO1861-conjugated nanoplexes have shown superior transfection efficiency and cell viability over supplementation of transfection medium with free SO1861. Targeted SO1861-equipped nanoplexes incorporating a targeting peptide were tested *in vitro* and *in vivo* in an aggressively growing neuroblastoma allograft model in mice. Using a suicide gene vector encoding the cytotoxic protein saporin, a slowed tumor growth and improved survival rate were observed for targeted SO1861-equipped nanoplexes compared to vehicle control.

## Introduction

Gene therapeutics, defined as biological drugs whose active ingredient consists of or contains a nucleic acid, are used to regulate, repair, replace, supplement, or inhibit a nucleic acid sequence.^1^ With close to 4000 gene therapy clinical trials that have been completed, are ongoing, or have been approved until March 2023, gene therapy has emerged as a promising approach for a variety of therapeutic indications, mainly cancer and monogenetic diseases.^2^ Nevertheless, more than 94% of all registered trials are in Phase I or II,^2^ and only 20 gene therapy medicinal products are currently approved by the U.S. Food & Drug Administration.^3^

All gene therapeutics approved to date use viral vectors, either integrating viral vectors such as retroviruses or lentiviruses for *ex vivo* transduction of autologous (patients’ own) cells or nonintegrating adeno-associated viruses (AAV) for the treatment of monogenetic diseases.^1,4^ In the latter case, the generation of neutralizing antibodies prevents the repeated administration of the same AAV-vector, making them suitable only for one-time therapy.^5^ Recently, nadofaragene firadenovec was approved as the first adenoviral vector-based gene therapy.^6^ Even though it is approved for repeated administration into the bladder, previous observations have shown severe immunogenicity upon repeated exposure to adenoviral vectors.^7^ Given the described limitations, as well as side effects and high costs prohibiting the widespread use of viral vectors,^8^ the further development and improvement of alternative, nonviral vectors for gene therapy approaches is of high relevance.^9^

Nucleic acids such as plasmid DNA carrying a transgene of interest are condensed by cationic polymers like poly-l-lysine (PLL), polyethylenimine, poly(amidoamine), or cationic lipids forming nanoparticles. The advantages of these alternative systems include a low immunogenicity and high packaging capacity. Furthermore, they are easily generated, even at large scale, and modifications to improve biodistribution, stability, and physicochemical properties are easy to implement. So far, compared to viruses, whose evolution has perfected gene delivery, nonviral vectors are less efficient in overcoming cellular barriers, resulting in significantly lower transfection efficiencies.^10 −13^

A major obstacle for efficient gene delivery using polymeric nanoparticles is the entrapment of the therapeutic cargo in endosomes and lysosomes, finally resulting in gene degradation.^14^ In previous work, we described the use of specific triterpene saponins isolated from plants of the Caryophyllaceae family—naturally occurring bidesmosidic glycosides consisting of a lipophilic triterpenoid (C_30_) aglycon and two branched hydrophilic sugar moieties—as endosomal escape enhancing agents for improved gene delivery of nanoparticles formed with PLL-derived peptides (also called nanoplexes).^15−17^ As previously demonstrated by single-cell analysis, the coadministration of saponins facilitates the escape of the entrapped gene from endosomes, thus creating a prerequisite for efficient gene delivery into the nucleus.^18^ The therapeutic potential of saponin-assisted transfection, which we termed sapofection,^16^ and its good tolerance were demonstrated *in vitro* and *in vivo* in an aggressively growing neuroblastoma model in mice.^17,19,20^

To date, gene-loaded nanoplexes and the endosomal escape enhancing saponin need to be administered separately aiming for suitable concentrations of both components at the target tissue at the same time. *In vitro*, this is easily achieved by adding both components to the cell culture medium in parallel, but harmonization of the administration routes *in vivo* proved to be more challenging. Intravenous (i.v.) injection of saponins results in massive hemolysis,^21^ so these were applied subcutaneously (s.c.) to the nuchal folds of mice. The nanoplexes, on the other hand, were injected i.v. into the tail vein. S.c. application of the saponin 1 h before i.v. injection of the nanoplexes proved to be an effective setup.^19,20^

**Figure 1:**
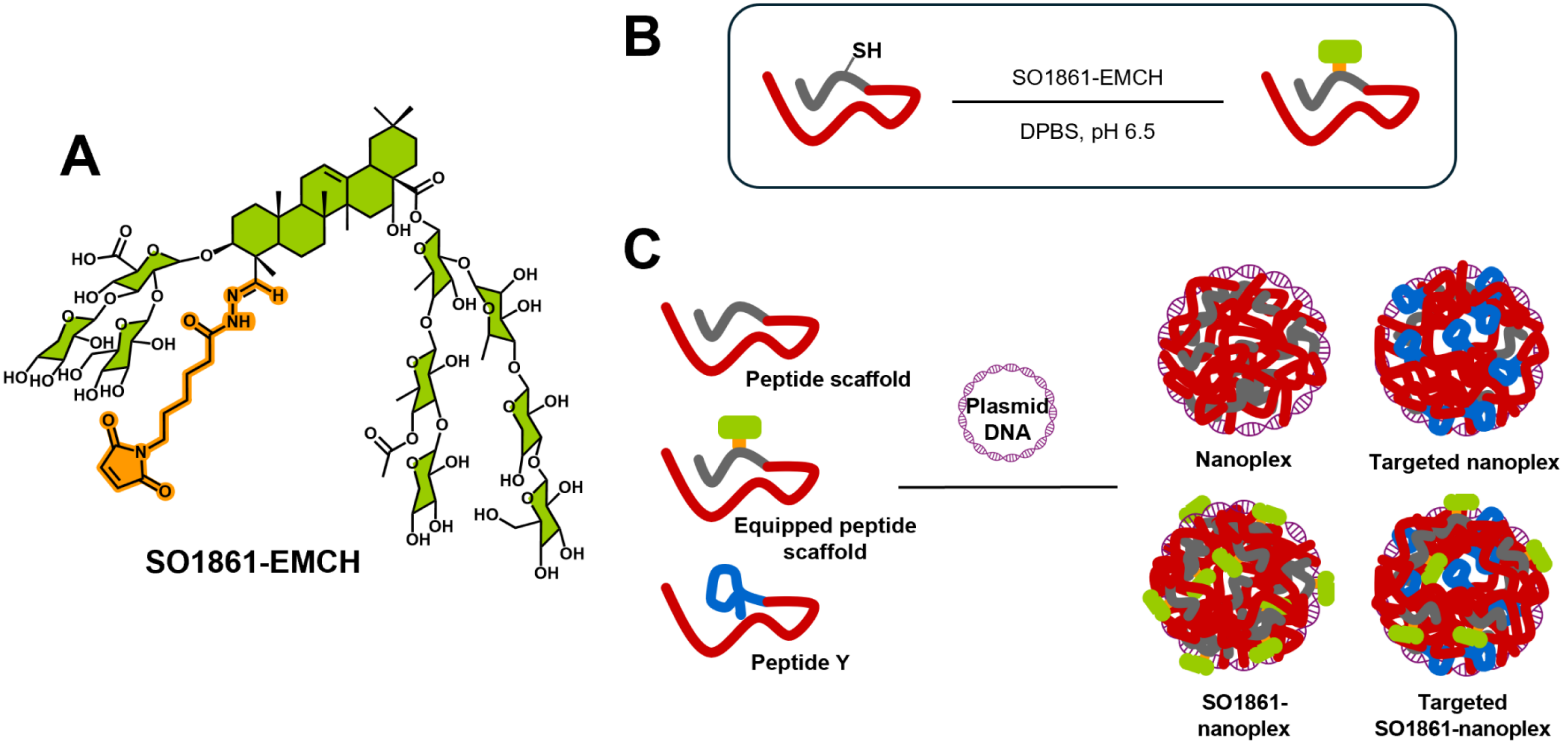
Structure of SO1861-EMCH (A). Michael addition between the maleimide of SO1861-EMCH and a single cysteine residue at the peptide scaffolds (B). Nanoplex formulation (C). Peptide scaffolds: red line represents K_16_-tail, gray line represents the additional amino acids and PEG spacer in peptides K_16_C and K_16_CPEG including a single cysteine residue, and blue line represents the targeting sequence of pepY (GACYGLPHKFCG).

Aiming to overcome the above limitations of state-of-the-art two-component sapofection systems, this work describes a gene therapy vector consisting of a plasmid DNA, a PLL-derived peptide, and the endosomal escape enhancing saponin SO1861^16^ as a single i.v. injectable formulation (Figure 1).

The central element of the chemical strategy designed for this purpose is a bifunctional *N*-ε-maleimidocaproic acid hydrazide (EMCH)-linker (Figure 1). The maleimide group of EMCH reacts in a Michael-type thiol-maleimide addition with a single cysteine residue included in the peptide PLL sequence to form a stable bond, while the hydrazide group reacts with the C-23 aldehyde of SO1861 to form a hydrazone bond that, being stable under physiological conditions, is rapidly hydrolyzed in the acidic endosomal and lysosomal compartments,^22^ allowing the intracellular release of SO1861.

## Results and Discussion

### SO1861-Equipped Peptide Scaffolds

With the aim of developing conditions for the Michael addition coupling between SO1861-EMCH and the cysteine residue at the PLL peptide, it was necessary to gather information about the relative stability of the hydrazone and maleimide functional groups. Although hydrazones are known to hydrolyze at pH *<* 7.0, this value for SO1861-EMCH was uncertain because of the hindered nature of the aldehyde at SO1861. Hence, the stability of SO1861-EMCH was studied in different buffers and pH values using ^1^H NMR (Figure S1). The observed hydrolysis rates indicated complete stability of both the maleimide and hydrazone groups in PBS at pH 7.4 and in 20 mM citrate at pH 6.0 for 24 h (Table S1).

Accordingly, reaction conditions for the thiol-maleimide conjugation were optimized in PBS at pH 6.5. This slightly acidic pH increases the thiol-versus-amine selectivity in the Michael-addition, allowing a selective conjugation at the single cysteine residue of the peptide scaffold without compromising the stability of the hydrazone and maleimide groups. Interestingly, under more acidic conditions mimicking the endolysosome (20 mM citrate, pH 4.5, 37 °C), the release of SO1861 was confirmed by recovery of the aldehyde proton by ^1^H NMR (Figure S1).

K_16_C and K_16_CPEG were designed as PLL-derived peptide scaffolds. Both include a K_16_-tail along with a single cysteine residue for selective SO1861-EMCH conjugation (Figure 1). In addition, K_16_CPEG carries a PEG_8_-linker intended to grant higher solubility and stability *in vivo* to the resulting nanoplexes. K_16_C and K_16_CPEG were functionalized with 0.25 and 0.5 equiv of SO1861-EMCH (Figure 1 and Supporting Information). These loadings were selected based on data previously obtained with the combined two-component sapofection approach. For all batches of equipped peptide scaffolds, successful conjugation of SO1861-EMCH to the peptide was confirmed using MALDI-MS (Figure S2). Purification via solid phase extraction (SPE) column was shown to be effective in the removal of free SO1861-EMCH as confirmed with very sensitive ESI-MS detection in the final product (Figure S3).

In the following, the SO1861-functionalized (equipped) peptide scaffolds are referred to as
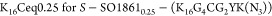

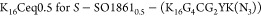

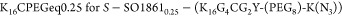

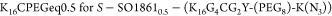
.

### Preparation and Characterization of Nanoplexes

**Figure 2:**
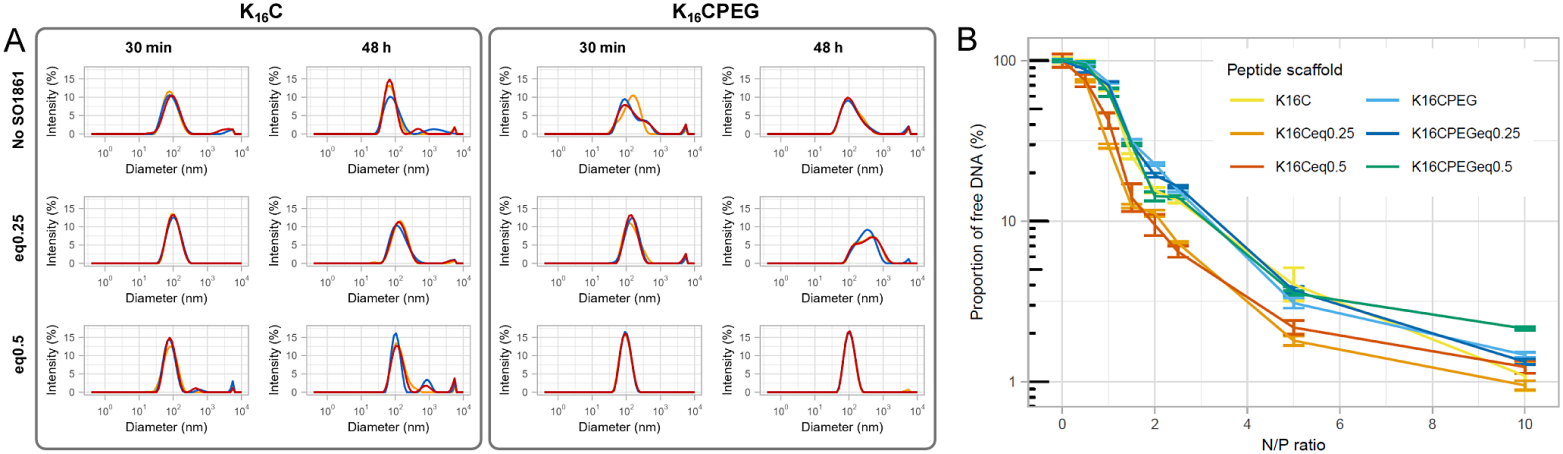
Size distribution of nanoplexes formulated with equipped and nonequipped peptide scaffolds after 30 min and 48 h (pEGFP-N3, N/P 10, 10 mM HEPES pH 7.1) (A). DNA complexation efficiency of equipped and nonequipped peptide scaffolds as a function of the N/P ratio (10 mM HEPES pH 7.1). Data are given as mean of triplicates, and error bars indicate standard deviation (B).

Dynamic light scattering (DLS) analysis of nanoplexes, produced by complexing 2.5 μg pEGFP-N3—a plasmid DNA vector encoding eGFP—with the equipped and nonequipped peptide scaffolds in 10 mM HEPES, pH 7.1 at N/P 10 revealed Z-averages, the intensity-weighted mean hydrodynamic size, of 80 to 160 nm 30 min after nanoplex formulation (Figure 2A). Interestingly, 200 nm is generally considered to be the upper size limit for clathrin-mediated endocytosis,^23^ a cutoff value not reached by any of the nanoplexes studied. The polydispersity index (PdI), indicating particle size distributions heterogeneity, varied between 0.1 and 0.4 (see Table 1). As seen in Figure 2A, for all nanoplexes, intensity-weighted size distributions showed a dominant peak at *D*_h_ around 100 nm. A minor share of aggregates with *D*_h_*>*1000 nm was detected for nanoplexes formulated with K_16_C, K_16_Ceq0.5, K_16_CPEG, and K_16_CPEGeq0.25. SO1861 conjugation was shown to reduce the mean size and heterogeneity of the nanoplexes, with 0.5 equiv showing superior results for K_16_CPEG and 0.25 equiv performing best for K_16_C (Figure 2A). Table 1 shows increased *D*_h_ and PdI for the nanoplexes formulated with equipped peptide scaffolds after a 48 h incubation period, indicating a swelling of the nanoplexes and increased aggregate formation over time. K_16_Ceq0.25- and K_16_CPEGeq0.5-nanoplexes exhibited the best stability, with *D*_h_*<*125 nm and PdI *<*0.25 after incubation for 48 h. Analysis of the nanoplexes in FBS-containing cell culture medium revealed complete stability for FBS concentrations ≤2.5% and a marginal size increase at 5% FBS (Figure S4).

**Table 1:** Hydrodynamic Diameter (*D*_h_), Polydispersity Index (PdI), and ζ-Potential of Nanoplexes Formulated with Equipped and Nonequipped Peptide Scaffolds (pEGFP-N3, N/P 10, 10 mM HEPES pH 7.1).^a^

**peptide scaffold**	**incubation time**	*D*_**h**_**(nm)**	**PdI**	**Z-potential (mV)**
K_16_C	30 min	86 *±* 5	0.28 ± 0.03	33 *±* 4
	48 h	80 *±* 3	0.32 ± 0.03	
K_16_Ceq0.25	30 min	97 *±* 1	0.16 ± 0.01	36 *±* 2
	48 h	124 *±* 1	0.24 ± 0.02	
K_16_Ceq0.5	30 min	117 *±* 23	0.28 ± 0.02	32 *±* 1
	48 h	186 *±* 11	0.31 ± 0.04	
K_16_CPEG	30 min	138 *±* 5	0.37 ± 0.01	29 *±* 3
	48 h	119 *±* 6	0.35 ± 0.02	
K_16_CPEGeq0.25	30 min	153 *±* 4	0.34 ± 0.02	30 *±* 1
	48 h	280 *±* 1	0.29 ± 0.01	
K_16_CPEGeq0.5	30 min	90 *±* 1	0.10 ± 0.01	31 *±* 1
	48 h	101 *±* 1	0.14 ± 0.02	

a Data are expressed as mean of triplicates ± standard deviation.

All investigated nanoplexes showed ζ-potentials, describing the value of electrostatic potential at the surface of hydrodynamic shear,^24^ around 30 mV, with slightly smaller values for the K_16_CPEG-derived peptide scaffolds (Table 1).

Figure 2B shows that all tested peptide scaffolds complexed DNA to a higher extent with rising N/P values. Complexation efficiency was slightly decreased due to the incorporation of the PEG_8_-spacer in the peptide K_16_CPEG, while the functionalization with SO1861 had no clear effect on DNA complexation. All investigated peptide scaffolds complexed *≥* 98% of DNA at N/P 10 in 10 mM HEPES pH 7.1.

### Transfection Efficiency *In Vitro*

**Figure 3:**
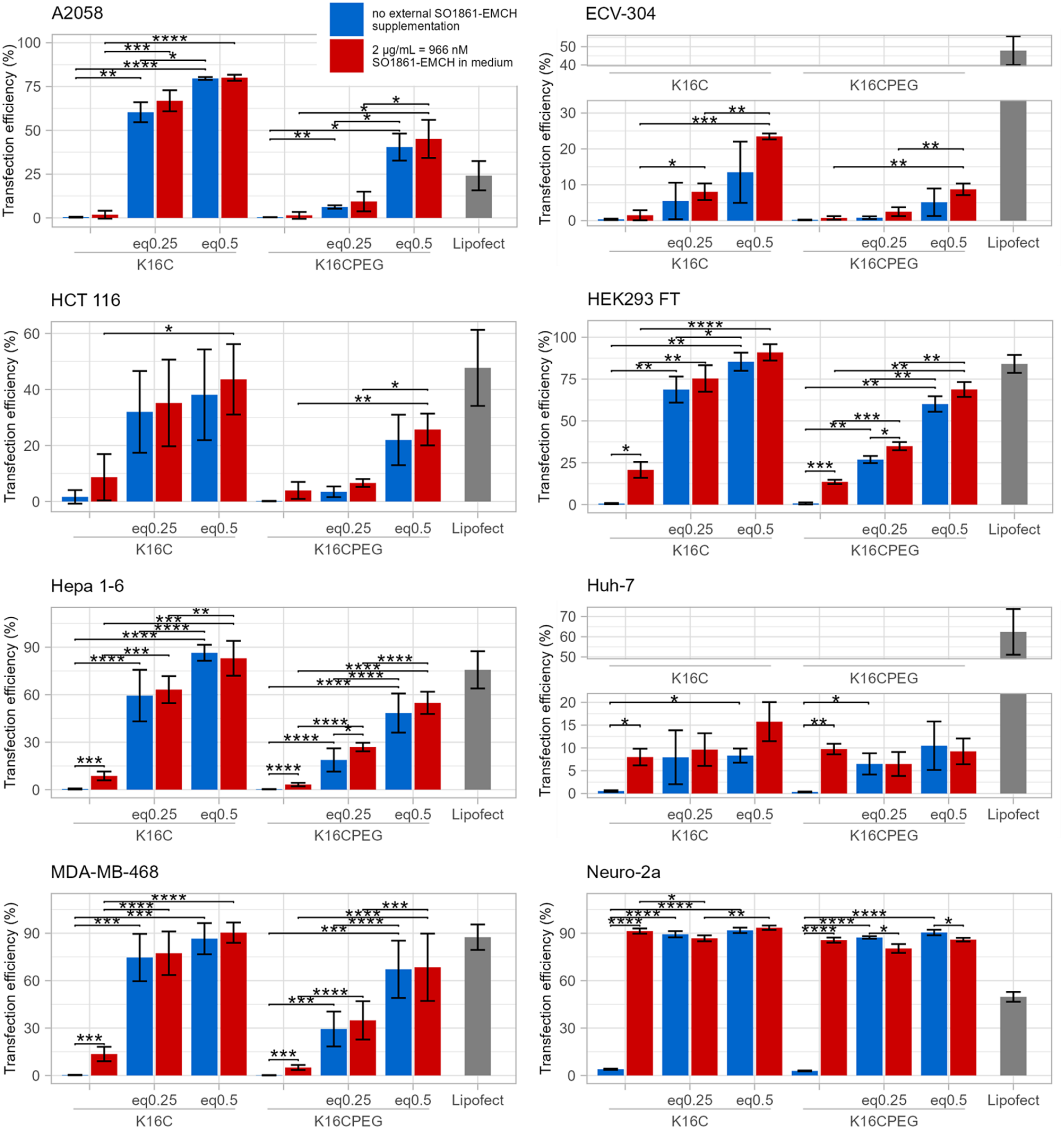
Transfection efficiency of nanoplexes (pEGFP-N3, N/P 10, 10 mM HEPES pH 7.1). Cells were incubated with nanoplexes and optionally with addition of external SO1861-EMCH for 48 h before transfection efficiency was determined using flow cytometry. Bar height indicates mean of three independent experiments (MDA-MB-468 and Hepa 1–6: triplicates, resulting in *n* = 9), and error bars show standard deviation. Significant differences were calculated with Student’s *t*-test (two-sided) except for differences in cell lines MDA-MB-468 and Hepa 1–6 that were calculated with Wilcoxon signed-rank test. *p *<*0.05, **p *<*0.01, ***p *<*0.001, ****p *<*0.0001.

The transfection efficiency of nanoplexes formulated with pEGFP-N3 and the equipped and nonequipped peptide scaffolds was determined in cell lines A2058 (human melanoma), ECV-304 (human urinary bladder carcinoma), HCT 116 (human colon carcinoma), HEK293 FT (human embryonic kidney cells), Hepa 1–6 (murine hepatoma), Huh-7 (human hepatoma), MDA-MB-468 (human adenocarcinoma), and Neuro-2a (murine neuroblastoma), representing a variety of tissues, organisms of origin and susceptibility to transfection. For all cell lines, Figure 3 shows that significant higher transfection efficiencies were achieved by nanoplexes formulated with equipped peptide scaffolds compared to their nonequipped analogs. This confirms the hypothesized transfection-enhancing capability of SO1861 when conjugated to nanoplexes.

The optimum loading of SO1861 in the peptide scaffolds seems to vary with the cell lines (blue bars in Figure 3). In A2058, ECV-304, HCT 116, HEK293 FT, Hepa 1–6, and MDA-MB-468 cells, the nanoplexes formulated with 0.5 equiv SO1861-equipped peptides transfected more efficiently than their counterparts formulated with 0.25 equiv, while no significant differences between the SO1861-loading of the peptide scaffolds were found for cell lines Huh-7 and Neuro-2a. The addition of further external SO1861-EMCH to the transfection medium (final concentration: 2 μg*/*mL, red bars in Figure 3), which was included to achieve maximum transfection efficiency, did not further improve transfection in the case of nanoplexes with conjugated SO1861, except for K_16_CPEGeq0.25-nanoplexes in HEK293 FT and Hepa 1–6 cells. In Neuro-2a cells, transfection efficiency was significantly decreased by the addition of external SO1861-EMCH to K_16_CPEGeq0.25- and K_16_CPEGeq0.5-nanoplexes.

In all cell lines, transfection efficiencies of the K_16_CPEG-derived peptide scaffolds were smaller than for their non-PEGylated analogs. This is in line with the slightly smaller cationic surface charge of the PEGylated nanoplexes (Table 1), which reduces their electrostatic interaction with the negatively charged cell membrane.^25^

The transfection efficiency of SO1861-equipped peptide scaffolds was comparable to that of Lipofectamine in cell lines HCT 116, HEK293 FT, Hepa 1–6, and MDA-MB-468. In A2058 and Neuro-2a cells, transfection efficiency of the equipped peptide scaffolds was higher than that of Lipofectamine, while it was lower in ECV-304 and Huh-7 cells.

**Figure 4:**
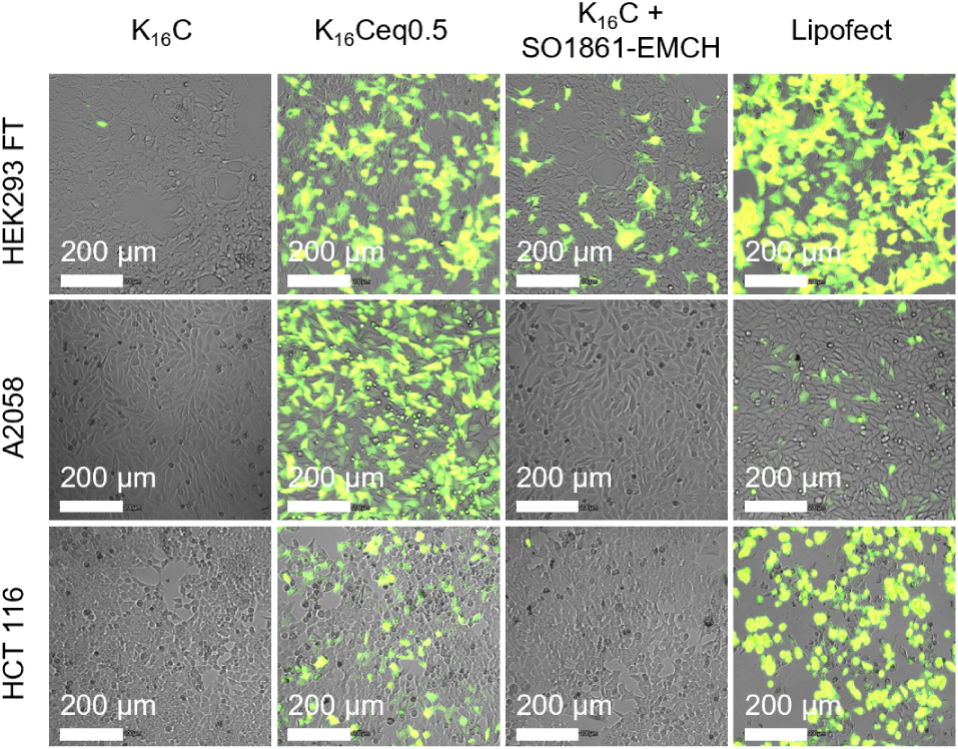
Fluorescence microscopy images of cell lines HEK293 FT, A2058, and HCT 116 after 48 h transfection with pEGFP-N3-nanoplexes. For K_16_C + SO1861-EMCH, 2 μg*/*mL SO1861-EMCH was supplemented to the transfection medium (SO1861-EMCH amount equals amount of conjugated SO1861-EMCH in K_16_Ceq0.5-nanoplexes). Pictures for each cell line were taken with constant exposure time, gain, and intensity.

The supplementation of 2 μg*/*mL SO1861-EMCH in the transfection medium equals the concentration of SO1861-EMCH in the K_16_Ceq0.5- and K_16_CPEGeq0.5-nanoplexes. The comparison of K_16_C + external SO1861-EMCH vs K_16_Ceq0.5 as well as K_16_CPEG + external SO1861-EMCH vs K_16_CPEGeq0.5 showed clearly increased transfection capabilities of the SO1861-equipped peptides in cell lines A2058, ECV-304, HCT 116, HEK293 FT, Hepa 1–6, and MDA-MB-468. Even to the untrained eye, the differences in transfection efficiency depicted in the fluorescence microscopy images shown in Figure 4 are strikingly obvious. The superiority of the SO1861-nanoplexes confirms the expected higher local concentration of SO1861 in the endosome. Furthermore, the conjugation of SO1861 to the nanoplex ensures colocalization of the plasmid DNA and SO1861 in the endosome. Thus, the endosomal escape enhancing effect of SO1861 is more likely to lead to a measurable result.

### Tolerability *In Vitro*

Impedance-based measurement of cell viability was used to investigate effects of the nanoplexes on cell growth during the 48 h transfection period in five different cell lines (Figure S6). Nanoplexes were formed with pEGFP-N3. Although no clear toxic effects were observed, a slightly slower cell growth was detected for Lipofectamine and transfections with K_16_Ceq0.5- and K_16_CPEGeq0.5-nanoplexes in some cell lines. In the case of the latter transfections, roughly 400 ng of covalently conjugated SO1861-EMCH per well were included in the nanoplexes. The observed slightly impaired cell viability does not seem to be primarily due to the amount of saponin, but rather to its covalent binding, since 1200 ng of external SO1861-EMCH supplemented in the cell culture medium without nanoplexes did not show a comparable effect. Given the higher transfection efficiency of SO1861 bound to the nanoplexes (*vide supra*), which can be explained by the higher concentration of SO1861 present locally in the endosome, the observed slightly increased toxicity is plausible. Since no toxic effect was observed for the use of 0.5 equiv of conjugated SO1861, the concentration range presented in this study appears to be a good guideline, with further increases of the amount of bound SO1861 seeming inadvisible in view of these results.

### Optimized Transfection with Targeted Nanoplexes

According to the observed superiority of conjugated SO1861 over external SO1861 *in vitro*, we envisioned the challenge to test the peptide-SO1861 conjugates in an *in vivo* setting. To achieve sufficient concentration in a tissue of interest while minimizing undesired effects on other tissues, a targeted delivery of the therapeutic cargo was considered for systemic application. Accordingly, we introduced the targeting peptide pepY into the nanoplexes. It consists of a DNA-complexing K_16_-tail, a GA-spacer and the targeting sequence YGLPHKF, which is cyclized by oxidation of two flanking cysteine residues (Figure 1).^26^ PepY has been shown to mediate targeted delivery of nucleic acids to cells of neuronal origin,^27,28^ human airway epithelium cells,^29,30^ primary vascular cells,^31^ and rabbit aorta cells.^32^ The transfection was shown to occur via a receptor-mediated mechanism, although the identity of the targeted receptor remains unclear.^26^ In previous work from our group, pepY has been successfully used for targeted delivery to murine neuroblastoma cell line Neuro-2a, both *in vitro* and *in vivo*.^19,20^

**Figure 5:**
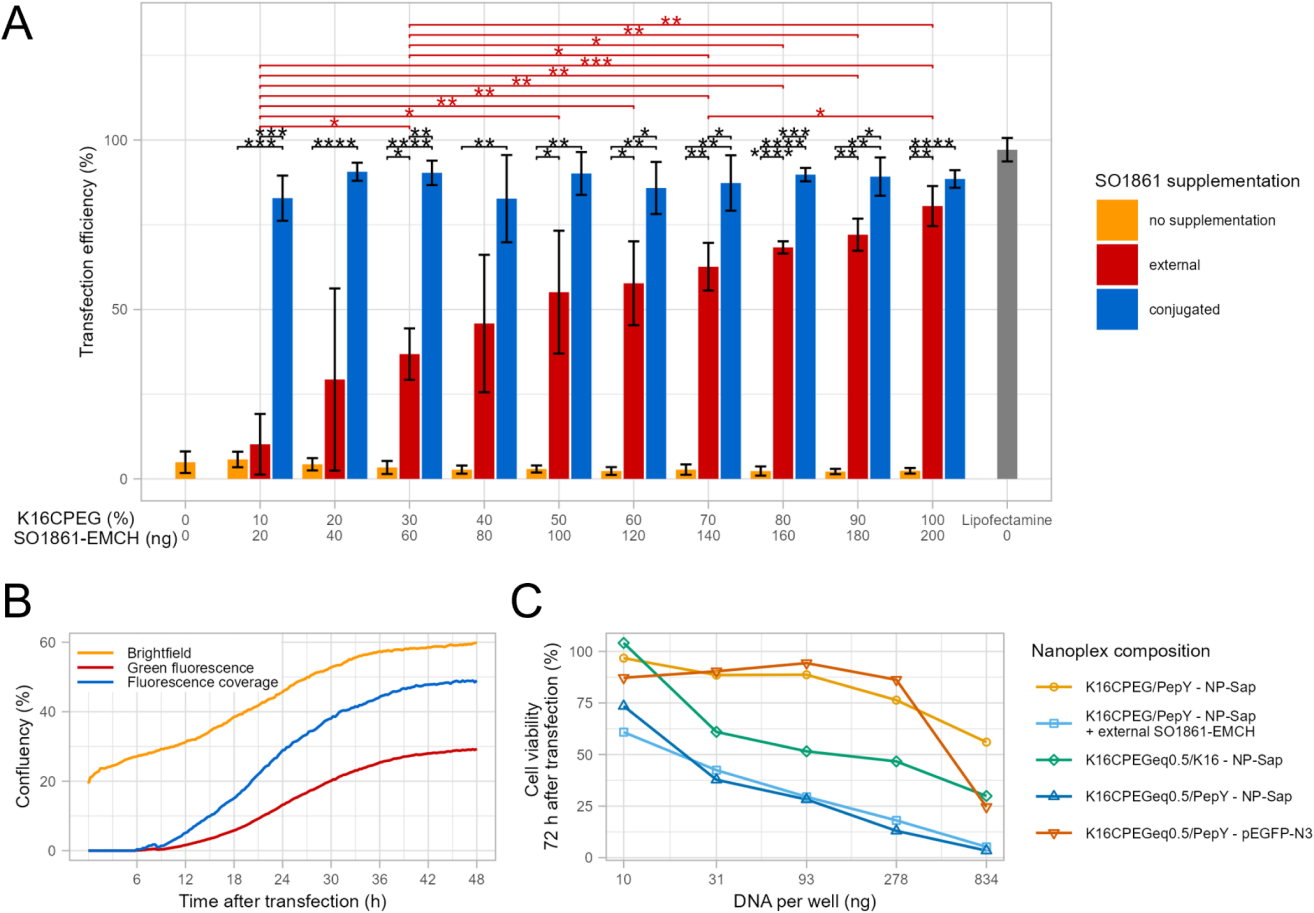
Transfection efficiency of targeted nanoplexes (NP-eGFP, N/P 10, HEPES-buffered mannitol pH 7.5) in Neuro-2a cell line. Cells were incubated with nanoplexes and optionally external SO1861-EMCH for 48 h before transfection efficiency was determined using flow cytometry. X axis labeling: upper panel gives the proportion (mol/mol) of peptides K_16_CPEG (for the yellow and red bars) or K_16_CPEGeq0.5 (for the blue bars), and lower panel states the absolute amount of SO1861-EMCH per well (same for externally supplemented and conjugated SO1861-EMCH). Bar height indicates mean of three independent experiments, and error bars show standard deviation. Significant differences were calculated with unpaired, two-sided Student’s *t*-test. *p *<*0.05, **p *<*0.01, ***p *<*0.001, ****p *<*0.0001 (A). Kinetic profile of eGFP expression in Neuro-2a cell line during transfection with targeted SO1861-nanoplexes (NP-eGFP complexed with 70% pepY and 30% K_16_CPEGeq0.5). Cell coverage (confluency) in the brightfield and green fluorescence (λex = 452 *±* 45 nm) were determined by the CytoSMART Cloud Service by means of image analysis. Fluorescence coverage indicates the share of eGFP-expressing cells (B). Dose–response curves of targeted nanoplexes (NP-Sap, N/P 10, HEPES-buffered mannitol pH 7.5) in Neuro-2a cell line. For the nanoplex formulation, the first specified peptide was used at 70% and the second at 30% (mol/mol). Cells were incubated with nanoplexes and optionally with addition of external SO1861-EMCH (amount equivalent to conjugated SO1861) for 72 h before cell viability was determined (MTS assay). Data are given as mean of three independent experiments with each of them performed in triplicate, resulting in *n* = 9 (C).

Targeted SO1861-nanoplexes were formulated by mixing the SO1861-equipped peptide scaffold K_16_CPEGeq0.5 and the targeting pepY before plasmid DNA was added and nanoplexes allowed to form. For optimization purposes, nanoplexes varying in their peptide composition were tested for their transfection efficiency *in vitro*. All nanoplexes were formulated with the Nanoplasmid vector NP-eGFP at N/P 10 with varying proportions (mol/mol) of the two peptide scaffolds. Nanoplasmid vectors are characterized by their minimalized bacterial origin, increased and prolonged plasmid-mediated transgene expression, and the absence of antibiotic resistance-encoding genes.^33^ Because of their regulatory compliance, nanoplasmid vectors were used for all *in vivo* studies. As control, targeted nanoplexes with equivalent peptide compositions, but lacking the covalently conjugated SO1861 were assessed in parallel, both with and without the supplementation of SO1861-EMCH in the transfection medium. Results are shown in Figure 5A. For transfections without saponin supplementation (yellow bars), decreasing transfection efficiencies from 4.9% for a nanoplex formulated exclusively with pepY to 2.4% for a nanoplex formulated exclusively with K_16_CPEG were observed with decreasing pepY content (not statistically significant). When SO1861-EMCH was supplemented in the cell culture medium in amounts equivalent to the conjugated amount of saponin in the respective SO1861-nanoplex (red bars), increasing transfection efficiencies from 10% for 20 ng SO1861-EMCH to 81% for 200 ng SO1861-EMCH in 100 μL well volume were observed, which is in line with previous observations of dose-dependent transfection-enhancing properties for triterpenoid saponins.^17^ Strikingly, when the same amount of SO1861 was covalently conjugated to the peptide scaffold (blue bars), transfection was significantly more efficient, with transfection efficiencies fluctuating between 82% and 91% for SO1861-EMCH amounts between 20 ng and 200 ng. This absence of a dose-dependent effect for conjugated SO1861 in the range of concentrations investigated points to a clear superiority of conjugated compared to externally supplemented SO1861. Indeed, comparatively lower amounts of conjugated SO1861-EMCH were sufficient to achieve maximum transfection efficiency in the Neuro-2a cell line.

Based on these *in vitro* transfection efficiencies and previous size and stability assessments, a targeted nanoplex formulated with 70% pepY and 30% K_16_CPEGeq0.5 (mol/mol) complexing NP-Sap, a plasmid vector encoding the cytotoxic ribosome-inactivating protein saporin,^34^ was chosen as treatment nanoplex for an *in vivo* antitumoral efficacy study. Continuous observation of the transfection of that targeted SO1861-nanoplex, using NP-eGFP, in Neuro-2a cell line, revealed eGFP-expressing cells as early as 5.5 h after intervention. The percentage of transfected cells increased continuously until 48 h after transfection (Figure 5B and Video S1).

To evaluate the effect of the pepY targeting peptide and SO1861 conjugation in the transfection of Neuro-2a cell line *in vitro*, dose–response curves of the treatment nanoplex (NP-Sap complexed with 70% pepY and 30% K_16_CPEGeq0.5 (mol/mol)) and corresponding controls were generated (Figure 5C). As saporin is a ribosome-inactivating protein leading to cell death, transfection efficiency was determined by measuring cell viability 72 h after transfection using MTS assay in comparison to an untreated control cell population. The comparison between the targeted SO1861-nanoplex (conjugated SO1861, dark blue line) and the targeted nonequipped nanoplex (yellow line) again confirmed the transfection-increasing effect of conjugated SO1861. This time, supplementation with equivalent amounts of external SO1861-EMCH in the transfection medium (light blue line) led to comparable effects as transfection with the SO1861-nanoplexes (dark blue line). The incorporation of targeting pepY instead of a nontargeted K_16_ peptide (green line) reduced cell viability by approximately 25% at all nanoplex concentrations examined, confirming the hypothesized nanoplex internalization enhancement by pepY. Transfection with the targeted SO1861 nanoplex formulated with the nontoxic eGFP-encoding plasmid pEGFP-N3 (red line) did not significantly reduce cell viability until the highest nanoplex concentration of 834 ng complexed DNA per well. Overall, these results confirm *in vitro* a synergistic effect of combining the pepY targeting peptide and SO1861 conjugation.

**Figure 6:**
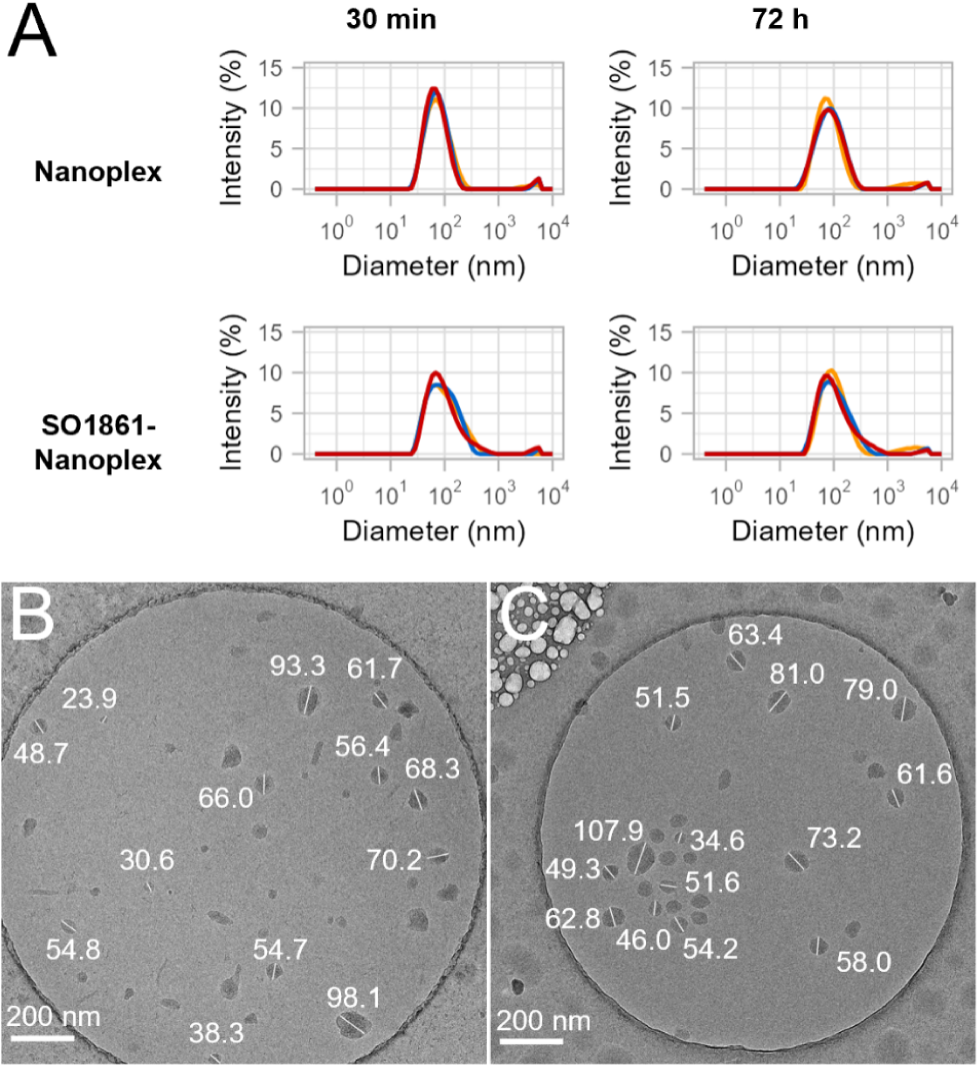
Size distribution of targeted nanoplexes formulated with 70% pepY and 30% K_16_CPEG for nanoplex or K_16_CPEGeq0.5 for SO1861-nanoplex (mol/mol) after 30 min and 72 h (NP-Sap, N/P 10, HEPES-buffered mannitol pH 7.5) (A). Cryo-TEM micrographs of targeted nanoplexes (B) and targeted SO1861-nanoplexes (C).

Before proceeding with the *in vivo* evaluation of the targeted SO1861-nanoplexes, they were characterized for their DNA complexation efficiency, size, size distribution, and surface charge, and these data compared with those from targeted (non-SO1861 equipped) nanoplexes as control. DNA complexation efficiency was determined to be 97% for both nanoplexes. DLS analysis revealed in both cases *D*_h_ well below 90 nm, with the targeted SO1861-nanoplex displaying a slightly larger size than the non-SO1861 equipped nanoplex. PdIs *<* 0.3 indicated a minimal extent of aggregate formation (Figure 6A and Table 2). After 72 h incubation at room temperature, both nanoplexes were only marginally enlarged. When these nanoplexes were analyzed by cryogenic transmission electron microscopy (Cryo-TEM), spherical particles with sizes between 30 and 100 nm were revealed (Figure 6B,C). Both nanoplexes exhibited moderately strong positive surface charge with ζ-potential values around 26 mV (Table 2).

**Table 2:** Hydrodynamic Diameter (*D*_h_), Polydispersity Index (PdI), and ζ-Potential of Targeted Nanoplexes Formulated with 70% PepY and 30% K_16_CPEG for Targeted Nanoplex or K_16_CPEGeq0.5 for Targeted SO1861-Nanoplex (mol/mol) (NP-Sap, N/P 10, HEPES-Buffered Mannitol pH 7.5).^a^

	**incubation**	*D*_**h**_**(nm)**	**PdI**	**Z-potential (mV)**
targeted nanoplex	30 min	68 *±* 1	0.23 *±* 0.01	26 *±* 1
	72 h	76 *±* 1	0.25 *±* 0.01	-
targeted SO1861-nanoplex	30 min	81 *±* 1	0.26 *±* 0.02	27 *±* 1
	72 h	91 *±* 2	0.27 *±* 0.01	-

a Data are expressed as mean of triplicates ± standard deviation.

### Antitumor Efficacy *In Vivo*

**Figure 7:**
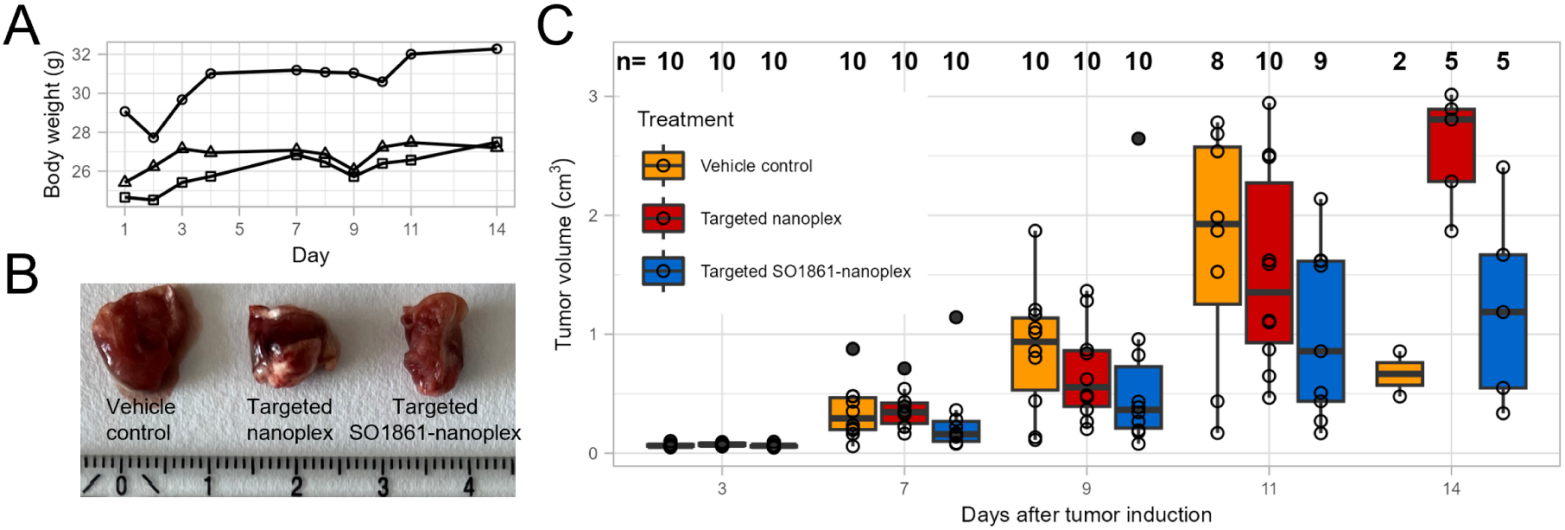
Body weight of three mice during tolerability study (A). Representative image of the isolated tumors from mice included in the antitumor efficacy study (B). Antitumor efficacy study: TV and survival rates on days 3, 7, 9, 11, and 14 after Neuro-2a tumor induction. Boxes of the boxplots represent the middle 50% of the data, the interquartile range (IQR). The horizontal line inside the box is the median. Whiskers represent minimum and maximum values. Any values lying outside 1.5 × IQR are plotted separately as filled dot. All data points were plotted as dots, and above each boxplot, the number of observations per group is given in bold, indicating survival rates. Decreasing group sizes over the course of the study were due to the termination of animals with tumor volumes *>*1.5 cm^3^ (C).

For the *in vivo* study, 6- to 8-week-old female NMRI nu/nu mice were used to test targeted nanoplexes for their tolerability and antitumor activity in the Neuro-2a neuroblastoma allograft model. The tolerability of the targeted SO1861-nanoplexes we first investigated in three nontumor-bearing mice. The application of the nanoplexes was performed analogously to the efficacy study. All three mice in the tolerance study did not show any sign of therapy-related side effects over the two-week study. Their body weight was stable with minimal fluctuations. Over the 2 weeks, all mice gained approximately 2 g in weight (Figure 7A); no losses in body weight *>*10% were observed. As massive hemolysis at the injection site was known for i.v. injection of free SO1861,^21^ the injection site was closely monitored during the study. Here, only a minimal short-term flush was observed at the injection sites, and no other severe alterations were noted. This indicated good tolerability of the intravenously administered targeted SO1861-nanoplexes in mice.

Antitumor efficacy of targeted SO1861-nanoplexes was investigated *in vivo* in an aggressive Neuro-2a neuroblastoma allograft model in mice, with targeted nonequipped nanoplexes and buffer as controls. Per group, 10 mice received five i.v. injections (100 μL with 30 μg complexed NP-Sap in the tail vein) each on days 1, 3, 5, 7, and 9 after s.c. tumor induction. Tumor volumes (TVs) were measured to determine treatment efficacy.

TV changes during the treatment clearly showed slowed tumor growth by treatment with targeted nanoplexes (red boxes), which was even slower for targeted SO1861-nanoplexes (blue boxes) compared to buffer only (vehicle control, yellow boxes) as depicted in Figure 7B,C7. After 9 days of tumor induction, tumors in the vehicle group had a mean volume of 0.87 cm^3^. Treatment with targeted nanoplexes resulted in a reduced mean TV of 0.68 cm^3^. Conjugation of SO1861 in the targeted SO1861-nanoplexes further reduced mean TV to 0.41 cm^3^ (excluding 1 confirmed outlier; mean TV including the outlier: 0.63 cm^3^). Treatment efficiency was also illustrated by improved survival rates in the treatment groups. Since the Neuro-2a allograft is an aggressively growing tumor model, untreated tumors reach the limiting size of *>*1.5 cm^3^ already 11 days after tumor induction. As a result, 80% of the animals from the placebo group had to be sacrificed for ethical reasons. The remaining animals exhibited a slow tumor growth over the whole study period, thereby indicating problems with tumor cell inoculation. With a survival rate of 50% after 14 days for both the targeted nanoplex and the targeted SO1861-nanoplex group, treatment was shown to prolong survival (Figure 7C).

The effect of nanoplex exposure on the major organs was investigated by analyzing the organ weights of the liver, spleen, and kidney of three randomly selected mice from each the vehicle control and the targeted SO1861-nanoplex group (Table 3). The markedly increased liver size observed in mice treated with targeted SO1861-nanoplexes in comparison to those receiving the vehicle control (600.4 ± 35.4 mg vs 326.1 ± 21.2 mg) can be attributed to Kupffer cell hyperplasia and inflammatory cell infiltration.

**Table 3:** Organ Weights of Mice Included in the *In Vivo* Efficacy Study. Liver, Spleen, and Kidney of Three Mice from Each the Vehicle Control Group (C1-3) and the Targeted SO1861-Nanoplex Receiving Treatment Group (T1-3) Were Removed and Immediately Frozen in Liquid Nitrogen.^a^

**organ**	**C1**	**C2**	**C3**	**T1**	**T2**	**T3**
liver	311.1	313.4	341.0	636.1	565.3	599.8
spleen	220.8	254.7	198.3	249.4	186.8	221.1
kidney	228.0	197.1	203.3	244.9	208.1	198.1

a After thawing, the organs were carefully dissected and weighed. Organ weights are given in milligrams.

Compared to the efficiency of the targeted SO1861-nanoplexes *in vitro*, the observed effect in the *in vivo* model appears to be somewhat smaller. This is not surprising assuming a possibly complex biodistribution of the i.v. injected particles. Also contributing to the observed lower efficacy is the high growth rate of the tumor, which might overrule the actual treatment effect. Thus, even with high transfection efficiencies, nontransfected cells remain unaffected and continue cell division leading to progressive tumor growth. Indeed, this effect is more pronounced in the evaluation after several days *in vivo* than in the *in vitro* evaluation after 48–72 h.

## Conclusions

Herein, we describe the successful preparation of covalent SO1861-peptide conjugates for nonviral gene delivery. The integration of SO1861 into nanoplexes containing plasmid DNA not only significantly increases the transfection efficiency of the parent nanoplex but also that of the previously practiced two-component sapofection approach, namely the separate application of the nanoplex and saponin component. We show this effect in numerous cell lines, which highlights the universal applicability of the developed SO1861-containing nanoplexes for transfecting cells of varying origin. The conjugation of SO1861 into the nanoplex eliminates the need to harmonize different biodistribution and application routes for two components, as it has been necessary in the sapofection routine practiced to date.

Targeted SO1861-equipped nanoplexes were produced by incorporation of the targeting peptide pepY. Targeted SO1861-nanoplexes bearing a suicide gene DNA encoding the cytotoxic protein saporin proved to be i.v. injectable and well-tolerated *in vivo* in mice. Effectiveness of these targeted SO1861-nanoplexes in an aggressive neuroblastoma allograft model *in vivo* corrobates the superiority of this nonviral gene delivery tool as a promising option for future gene therapy on cancer diseases. As exchange of the therapeutic DNA vector is easy to implement in the described nanoplexes, this strategy is also of interest for other indications treatable by gene therapeutic approaches.

## Experimental Section

### Preparation of Peptide-SO1861 Conjugates

Peptides K_16_C [K_16_G_4_CG_2_YK(N_3_), MW 2830.6], K_16_CPEG [K_16_G_4_CG_2_Y-(PEG_8_)-K(N_3_), MW 3254.1], K_16_ [K_16_, MW 2067.8], and peptide Y [pepY, K_16_GACYGLPHKFCG, MW 3302.2] (sequence indicated from *N*- to C-terminus, C-terminus is amidated, K(N_3_) denotes a lysine whose ε-amino group is exchanged for an azide group) were acquired with *≥* 80% purity from GeneCust, France. Peptides were received in lyophilized form, dissolved in ultrapure (σ *≤* 0.055 μS*/*cm, LaboStar, Siemens AG, Germany), sterile-filtered water, and stored at *–*20 ^◦^C in aliquots. Isolated and purified SO1861 from *Saponaria officinalis* L., functionalized with an EMCH linker at the C-23 carbonyl group (SO1861-EMCH, MW 2071.1), was supplied by Sapreme Technologies, The Netherlands. For the preparation of peptide-SO1861 conjugates, peptides K_16_C and K_16_CPEG were dissolved at 2 mg*/*mL and SO1861-EMCH was dissolved at 1 mg*/*mL in Dulbecco’s Phosphate-Buffered Saline (DPBS), pH 6.5 immediately before starting the reaction. In a 15 mL conical tube, 5 mL of the peptide solution was mixed with the appropriate amount of SO1861-EMCH-solution (0.25 or 0.5 equiv, mol/mol) and diluted with DPBS, pH 6.5 to a final volume of 10 mL [e.g., for K_16_Ceq0.25:5 mL K_16_C (2 mg*/*mL in DPBS, pH 6.5), 1.829 mL SO1861-EMCH (1 mg*/*mL in DPBS, pH 6.5), 3.171 mL DPBS, pH 6.5). The reaction mixture was incubated for 16 h under orbital shaking (800 rpm) at room temperature. Then, the reaction solution was transferred to dialysis tubes (Ready Lyzer 10, MWCO 1 kDaA, SERVA Electrophoresis GmbH, Germany) and dialyzed against ultrapure water for 24 h at 8 ^◦^C. The dialysis buffer was exchanged twice. The dialyzate was lyophilized. The resulting peptide-SO1861 conjugates are hereafter referred to as equipped peptide scaffolds (Figure 1).To ensure the absence of any free unreacted SO1861-EMCH, batches that were produced for *in vivo* studies were subjected to solid phase extraction after dialysis and before lyophilization of the final product. A CHROMABOND HR-XAW SPE column (3 mL/60 mg) was conditioned with 5 mL methanol (HiPerSolv CHROMANORM, VWR, USA), followed by 5 mL ultrapure water before the dialyzate was loaded to the column. Afterward, the column was washed with 2 mL ultrapure water. The flow-through of sample loading and the following washing step were collected and pooled before lyophilization.

### Nanoplex Formulation

The nanoplasmid vectors NTC9385R-Sap-BGH pA (NP-Sap, 2585 bp) encoding the cytotoxic ribosome-inactivating protein saporin and NTC9385R-EGFP-BGH pA (NP-eGFP, 2487 bp) coding for enhanced green fluorescent protein (eGFP) were produced by Nature Technology Corporation, USA. These Nanoplasmid vectors are characterized by their minimalized bacterial origin, increased and prolonged plasmid-mediated transgene expression, and the absence of antibiotic resistance-encoding genes. Because of their regulatory compliance, Nanoplasmid vectors were used for all *in vivo* studies. For exploratory work and optimization steps, pEGFP-N3 (GenBank Accession: U57609, 4729 bp), a plasmid DNA vector also encoding eGFP, was amplified in DH5α-*Escherichia coli* bacteria (Thermo Fisher Scientific, USA) and isolated and purified using QIAGEN Plasmid Mega Kit (Qiagen, Germany) according to the manufacturer protocol.

Upon mixing with peptide scaffolds, which are positively charged at physiological pH due to their K_16_-tail, nucleic acids are complexed leading to nanoplexes. Nanoplex composition is characterized by its N/P ratio, which is the molar ratio of charged nitrogen atoms (introduced by the protonated amino groups in the lysine side chains of the peptides) and charged phosphate groups (introduced by the backbone of the plasmid DNA).

For nanoplex formation, equal volumes of peptide and DNA solutions were mixed by adding the DNA to the peptide followed by rapidly pipetting the resulting solution up and down 20 times. The concentration of the DNA solution and the used volumes are given for the various applications in the corresponding descriptions.

For all *in vitro* and characterization experiments of equipped and nonequipped peptide scaffolds, 10 mM HEPES pH 7.1 (PUFFERAN CELLPURE *≥* 99.5%, Carl Roth GmbH + Co. KG, Germany) was used as nanoplex formulation buffer. Isotonic HEPES-buffered mannitol solution [HBM, 270 mM D-mannitol (low endotoxin pharma grade, PanReac AppliChem ITW Reagents, Germany), 5 mM HEPES pH 7.5] was used as nanoplex formulation buffer for all experiments with targeted nanoplexes and for the *in vivo* study. For visualization with electron microscopy, nanoplexes were formulated in ultrapure, sterile-filtered water.

The nanoplex solutions were incubated for 30 min at room temperature and then, either used directly or diluted with buffer or cell culture medium, according to the experiment setup.

For the formulation of targeted nanoplexes, which include the incorporation of two different peptides, these were mixed prior to the addition of the DNA solution. The proportions described of the two peptides relate to their molar ratio. For example, the composition of 70% pepY/30% K_16_CPEGeq0.5 targeted nanoplex tested *in vivo* (N/P 10) refers to pepY accounting for N/P 7 plus K_16_CPEGeq0.5 for N/P 3.

### DNA Complexation Efficiency

DNA complexation efficiency was assessed using a highly selective, double-stranded (ds) DNA-binding fluorescent dye to quantify the amount of free plasmid DNA after nanoplex formulation. For each nanoplex, 400 ng DNA were complexed in a total volume of 20 μL. QuantiFluor dsDNA Dye (Promega GmbH, Germany) was diluted 1:400 in the nanoplex formulation buffer, and 200 μL of the resulting solution was transferred to each well of a black 96-well microtiter plate (Greiner-Bio-One GmbH, Germany). 5 μL of the incubated nanoplex solutions was added per well, which equals a total amount of 100 ng DNA per well. For the blank, the same volume of formulation buffer was used. Each sample was measured in triplicate. Fluorescence intensity was measured using a microplate reader (TECAN infinite F200, Switzerland). After mixing the solutions for 5 min by orbital shaking at 300 rpm, the plate was incubated for another 5 min at room temperature in the dark. Fluorescence measurement was performed with λ_ex_ = 504 nm and λ_em_ = 531 nm; the gain was set to optimal. Fluorescence intensity signals were corrected by the signal of the blank buffer sample. The signal for nanoplexes formulated at N/P 0 was set to 100% of free DNA and the amount of free DNA for the other samples was calculated by fluorescence intensity (NP x)/fluorescence intensity (NP 0) × 100%, as the assay is linear over a range of 0.05 ng to 200 ng of dsDNA input.

### Transfection Efficiency *In Vitro*

To investigate the transfection efficiency *in vitro*, cell suspensions acquired during routine passaging were counted using a Neubauer counting chamber. Cells were seeded into clear 96-well plates (CELLSTAR TC, Greiner Bio-One GmbH, Germany) using a culture volume of 100 μL per well. 5000 cells were seeded for the cell lines A2058, ECV-304, HCT 116, and Neuro-2a, 7500 for HEK293 FT cell line, and 10 000 for the cell lines Huh-7, Hepa 1–6 and MDA-MB-468. Cells were incubated under the regular culture conditions for 24 h before the complete culture medium was exchanged with new culture medium (including FBS) supplemented with nanoplexes (formulated as described above) and optionally 2 μg*/*mL external SO1861-EMCH. Lipofect (Lipofectamine 3000, Thermo Fisher Scientific, USA) transfections, that were performed in parallel for comparison, were prepared accordingly using the same buffer as for the nanoplex formulation. 100 ng of complexed plasmid DNA was used per well and cells were incubated with the nanoplex-containing media for 48 h (pEGFP-N3- and NP-eGFP transfections) or 72 h (NP-Sap transfections) using the regular cultivation conditions. A CytoSMART Lux3 FL, a small fluorescence live-cell imaging microscope was used to monitor cell growth and eGFP-expression during the 48 h incubation period. Cell coverage (confluency) in the brightfield and green fluorescence (λex = 452 *±* 45 nm) channel were determined by the CytoSMART Cloud Service by means of image analysis. Fluorescence coverage indicating the share of eGFP-expressing cells was calculated by confluency green fluorescence (%)/confluency brightfield (%)×100%.

For the quantification of transfection efficiency, cells were detached using Trypsin/Versene (Lonza Group, Switzerland). The resulting cell suspensions were kept on ice until analysis by flow cytometry using a CytoFLEX S (Beckmann Coulter GmbH, Germany) flow cytometer. To ensure a reproducible evaluation and quantitative statement, only single, intact cells were included in the analysis. These were gated based on their forward scatter (FSC). The gate was established manually using blank (untreated) cell suspensions. As shown in Figure S5A, the peak width was plotted against the peak height of the forward scatter signal in a dot plot and the population of singlets was selected manually. Cell debris exhibits both lower signal heights and widths, a broadening of the signal with no change in height occurs with groups of cells. A minimum number of 5000 single cells were included in the analysis of each condition. For the evaluation of eGFP-expression, peak height of the fluorescence signal in the fluorescein isothiocyanate (FITC)-channel (excitation with blue laser λ = 488 nm; emission band-pass filter λ = 525 ± 40 nm) was determined. As depicted in Figure S5B, all cells exhibiting eGFP-related fluorescence signals in the FITC-channel above the threshold set by the blank cell population were considered transfected. The transfection efficiency in percent indicates the proportion of transfected singlets of all singlets measured.

For NP-Sap transfection, transfection efficiency was determined by assessing cell viability 72 h after transfection using MTS assay (CellTiter 96 AQ_ueous_ One Solution Cell Proliferation Assay, Promega GmbH, Germany) according to the manufacturer protocol using an incubation time of 2 h. Each *in vitro* transfection experiment was performed independently three times.

### *In Vivo* Investigations

For the *in vivo* study, 6- to 8-week-old female NMRI nu/nu mice were used to test targeted nanoplexes for their tolerability and antitumor activity in the Neuro-2a neuroblastoma allograft model. All animal experiments were performed in accordance with the United Kingdom Coordinated Committee on Cancer Research (UKCCR) guidelines and were approved by the responsible local authorities (State Office of Health and Social Affairs, Berlin, Germany; approval No. G03333/18 and Reg0010/19). Treatment nanoplexes (NP-Sap complexed with 70% pepY and 30% K_16_CPEGeq0.5, hereinafter referred to as targeted SO1861-nanoplex) were compared to nonequipped nanoplexes (NP-Sap complexed with 70% pepY and 30% K_16_CPEG, hereinafter referred to as targeted nanoplex) and vehicle control as placebo.

### Tolerability Studies

The toxicity of the targeted SO1861-nanoplexes was assessed in three NMRI nu/nu female mice without tumor induction. Mice were injected i.v. with targeted SO1861-nanoplexes (30 μg complexed NP-Sap in 100 μL) every 2 days with a total number of five injections. Mice were monitored for condition and for potential side effects, including skin reactions (flush) at the injection site. Body weight was measured every 2 days for 2 weeks.

### Antitumor Efficacy

1 ×10^6^ Neuro-2a cells in DPBS were injected s.c. in the left flank of NMRI nu/nu mice to induce neuroblastoma tumors. Thirty animals were then randomly allocated to the three treatment groups (*n* = 10 mice/group). Injection schedule was the same for all groups: a total of 5 injections were administered i.v. on days 1, 3, 5, 7, and 9 after tumor induction. The control group received 100 μL HBM as vehicle control, the targeted nanoplex group received 100 μL targeted nanoplex in HBM (30 μg complexed NP-Sap per injection), and the targeted SO1861-nanoplex group received 100 μL targeted SO1861-nanoplex in HBM (30 μg complexed NP-Sap per injection). Tumor size and body weight were determined twice a week during the study period. The treatment efficacy was determined by measurement of tumor volumes (TV). TV measurement was performed with a digital caliper and TVs were calculated using the formula TV = 1/2 × length × width^2^. Studies were terminated for ethical reasons when animals reached a TV *>* 1.5 cm^3^.

## Supporting Information Available

Stability analysis of SO1861-EMCH by ^1^H NMR, additional characterization of peptide scaffolds and polyplexes, additional cell experiments, and statistical analysis (PDF)
Time-lapse video of transfection (MP4)

## References

[ref1] Kulkarni J. A.; Witzigmann D.; Thomson S. B.; Chen S.; Leavitt B. R.; Cullis P. R.; van der Meel R. The current landscape of nucleic acid therapeutics. Nat. Nanotechnol. 2021, 16, 630–643. 10.1038/s41565-021-00898-0. 34059811

[ref2] The Journal of Gene Medicine, Gene Therapy Clinical Trials Worldwide, https://a873679.fmphost.com/fmi/webd/GTCT. 2024.

[ref3] U.S. Food & Drug Administration, Approved Cellular and Gene Therapy Products, https://www.fda.gov/vaccines-blood-biologics/cellular-gene-therapy-products/approved-cellular-and-gene-therapy-products. 2024.

[ref4] Bulcha J. T.; Wang Y.; Ma H.; Tai P. W. L.; Gao G. Viral vector platforms within the gene therapy landscape. Signal Transduction Targeted Ther. 2021, 6 (1), 53 10.1038/s41392-021-00487-6. PMC7868676 33558455

[ref5] Burdett T.; Nuseibeh S. Changing trends in the development of AAV-based gene therapies: a meta-analysis of past and present therapies. Gene Ther. 2023, 30, 323–335. 10.1038/s41434-022-00363-0. 36089633

[ref6] U.S. Food & Drug Administration, Adstiladrin, https://www.fda.gov/vaccines-blood-biologics/cellular-gene-therapy-products/adstiladrin. 2024.

[ref7] Shirley J. L.; De Jong Y. P.; Terhorst C.; Herzog R. W. Immune Responses to Viral Gene Therapy Vectors. Mol. Ther. 2020, 28, 709–722. 10.1016/j.ymthe.2020.01.001. 31968213 PMC7054714

[ref8] Gene therapy at the crossroads. Gene therapy at the crossroads. Nat. Biotechnol. 2022, 40, 621 621. 10.1038/s41587-022-01346-7. 35534558

[ref9] Sheridan C. Why gene therapies must go virus-free. Nat. Biotechnol. 2023, 41, 737–739. 10.1038/s41587-023-01824-6. 37316735

[ref10] Freitag F.; Wagner E. Optimizing synthetic nucleic acid and protein nanocarriers: The chemical evolution approach. Adv. Drug Delivery Rev. 2021, 168, 30–54. 10.1016/j.addr.2020.03.005. 32246984

[ref11] Kumar R.; Santa Chalarca C. F.; Bockman M. R.; Bruggen C. V.; Grimme C. J.; Dalal R. J.; Hanson M. G.; Hexum J. K.; Reineke T. M. Polymeric Delivery of Therapeutic Nucleic Acids. Chem. Rev. 2021, 121, 11527–11652. 10.1021/acs.chemrev.0c00997. 33939409

[ref12] Sayed N.; Allawadhi P.; Khurana A.; Singh V.; Navik U.; Pasumarthi S. K.; Khurana I.; Banothu A. K.; Weiskirchen R.; Bharani K. K. Gene therapy: Comprehensive overview and therapeutic applications. Life Sci. 2022, 294, 120375 10.1016/j.lfs.2022.120375. 35123997

[ref13] Sahu K. K.; Pradhan M.; Singh D.; Singh M. R.; Yadav K. Non-viral nucleic acid delivery approach: A boon for state-of-the-art gene delivery. J. Drug Del. Sci. Technol. 2023, 80, 104152 10.1016/j.jddst.2023.104152.

[ref14] Gilleron J.; Querbes W.; Zeigerer A.; Borodovsky A.; Marsico G.; Schubert U.; Manygoats K.; Seifert S.; Andree C.; Stöter M.; et al. Image-based analysis of lipid nanoparticle–mediated siRNA delivery, intracellular trafficking and endosomal escape. Nat. Biotechnol. 2013, 31, 638–646. 10.1038/nbt.2612. 23792630

[ref15] Sama S. Unstersuchung von Saponinen als neuartige Verstärker der Transfektion. Freie universität berlin repository, 2018. 10.17169/refubium-15.

[ref16] Sama S.; Jerz G.; Schmieder P.; Woith E.; Melzig M. F.; Weng A. Sapofectosid – Ensuring non-toxic and effective DNA and RNA delivery. Int. J. Pharm. 2017, 534 (1–2), 195–205. 10.1016/j.ijpharm.2017.10.016. 29054027

[ref17] Clochard J.; Jerz G.; Schmieder P.; Mitdank H.; Tröger M.; Sama S.; Weng A. A new acetylated triterpene saponin from Agrostemma githago L. modulates gene delivery efficiently and shows a high cellular tolerance. Int. J. Pharm. 2020, 589, 119822 10.1016/j.ijpharm.2020.119822. 32861772

[ref18] Weng A.; Manunta M. D. I.; Thakur M.; Gilabert-Oriol R.; Tagalakis A. D.; Eddaoudi A.; Munye M. M.; Vink C. A.; Wiesner B.; Eichhorst J.; et al. Improved intracellular delivery of peptide- and lipid-nanoplexes by natural glycosides. J. Controlled Release 2015, 206, 75–90. 10.1016/j.jconrel.2015.03.007. 25758332

[ref19] Sama S.; Woith E.; Walther W.; Jerz G.; Chen W.; Hart S.; Melzig M. F.; Weng A. Targeted suicide gene transfections reveal promising results in nu/nu mice with aggressive neuroblastoma. J. Controlled Release 2018, 275, 208–216. 10.1016/j.jconrel.2018.02.031. 29481823

[ref20] Mitdank H.; Tröger M.; Sonntag A.; Shirazi N. A.; Woith E.; Fuchs H.; Kobelt D.; Walther W.; Weng A. Suicide nanoplasmids coding for ribosome-inactivating proteins. Eur. J. Pharm. Sci. 2022, 170, 106107 10.1016/j.ejps.2021.106107. 34958884

[ref21] Gilabert-Oriol R.; Mergel K.; Thakur M.; von Mallinckrodt B.; Melzig M. F.; Fuchs H.; Weng A. Real-time analysis of membrane permeabilizing effects of oleanane saponins. Bioorg. Med. Chem. 2013, 21, 2387–2395. 10.1016/j.bmc.2013.01.061. 23454223

[ref22] Greenfield R. S.; Kaneko T.; Daues A.; Edson M. A.; Fitzgerald K. A.; Olech L. J.; Grattan J. A.; Spitalny G. L.; Braslawsky G. R. Evaluation in Vitro of Adriamycin Immunoconjugates Synthesized Using an Acid-sensitive Hydrazone Linker. Cancer Res. 1990, 50, 6600–6607. 2208122

[ref23] Rejman J.; Oberle V.; Zuhorn I. S.; Hoekstra D. Size-dependent internalization of particles via the pathways of clathrin- and caveolae-mediated endocytosis. Biochem. J. 2004, 377, 159–169. 10.1042/bj20031253. 14505488 PMC1223843

[ref24] Lowry G. V.; Hill R. J.; Harper S.; Rawle A. F.; Hendren C. O.; Klaessig F.; Nobbmann U.; Sayre P.; Rumble J. Guidance to improve the scientific value of zeta-potential measurements in nanoEHS. Environ. Sci.: nano 2016, 3, 953–965. 10.1039/C6EN00136J.

[ref25] Asati A.; Santra S.; Kaittanis C.; Perez J. M. Surface-Charge-Dependent Cell Localization and Cytotoxicity of Cerium Oxide Nanoparticles. ACS Nano 2010, 4, 5321–5331. 10.1021/nn100816s. 20690607 PMC2947560

[ref26] Writer M. J.; Marshall B.; Pilkington-Miksa M. A.; Barker S. E.; Jacobsen M.; Kritz A.; Bell P. C.; Lester D. H.; Tabor A. B.; Hailes H. C.; et al. Targeted Gene Delivery to Human Airway Epithelial Cells with Synthetic Vectors Incorporating Novel Targeting Peptides Selected by Phage Display. J. Drug Target. 2004, 12, 185–193. 10.1080/10611860410001724459. 15506167

[ref27] Tagalakis A. D.; He L.; Saraiva L.; Gustafsson K. T.; Hart S. L. Receptor-targeted liposome-peptide nanocomplexes for siRNA delivery. Biomaterials 2011, 32, 6302–6315. 10.1016/j.biomaterials.2011.05.022. 21624650

[ref28] Tagalakis A. D.; Saraiva L.; McCarthy D.; Gustafsson K. T.; Hart S. L. Comparison of Nanocomplexes with Branched and Linear Peptides for SiRNA Delivery. Biomacromolecules 2013, 14, 761–770. 10.1021/bm301842j. 23339543

[ref29] Tagalakis A. D.; McAnulty R. J.; Devaney J.; Bottoms S. E.; Wong J. B.; Elbs M.; Writer M. J.; Hailes H. C.; Tabor A. B.; O’Callaghan C.; et al. A Receptor-targeted Nanocomplex Vector System Optimized for Respiratory Gene Transfer. Mol. Ther. 2008, 16, 907–915. 10.1038/mt.2008.38. 18388925

[ref30] Manunta M. D. I.; McAnulty R. J.; Tagalakis A. D.; Bottoms S. E.; Campbell F.; Hailes H. C.; Tabor A. B.; Laurent G. J.; O’Callaghan C.; Hart S. L. Nebulisation of Receptor-Targeted Nanocomplexes for Gene Delivery to the Airway Epithelium. PLoS One 2011, 6, e26768 10.1371/journal.pone.0026768. 22046351 PMC3202583

[ref31] Irvine S. A.; Meng Q.-H.; Afzal F.; Ho J.; Wong J. B.; Hailes H. C.; Tabor A. B.; McEwan J. R.; Hart S. L. Receptor-targeted Nanocomplexes optimized for Gene Transfer to Primary Vascular Cells and Explant Cultures of Rabbit Aorta. Mol. Ther. 2008, 16, 508–515. 10.1038/sj.mt.6300381. 18180778

[ref32] Meng Q. H.; Irvine S.; Tagalakis A. D.; McAnulty R. J.; McEwan J. R.; Hart S. L. Inhibition of neointimal hyperplasia in a rabbit vein graft model following non-viral transfection with human iNOS cDNA. Gene Ther. 2013, 20, 979–986. 10.1038/gt.2013.20. 23636244 PMC3795475

[ref33] Tiwari N.; Beilowitz J.; Sampson C.; Peterson D.; Carnes A.; Williams J. Production of a Nanoplasmid with a large gene insert using the HyperGRO fermentation process. Vaccine Technology VI 2016, 10.1016/S1525-0016(16)33520-1.

[ref34] Weng A.; Thakur M.; von Mallinckrodt B.; Beceren-Braun F.; Gilabert-Oriol R.; Wiesner B.; Eichhorst J.; Böttger S.; Melzig M. F.; Fuchs H. Saponins modulate the intracellular trafficking of protein toxins. J. Controlled Release 2012, 164, 74–86. 10.1016/j.jconrel.2012.10.002. 23063550

