# Supplementary material for: Broadening the Scope of Sapofection: Cationic Peptide-Saponin Conjugates Improve Gene Delivery In Vitro and In Vivo
Source: ACS Appl Mater Interfaces. 2024 Jul 6;16(28):36095–105. doi: 10.1021/acsami.4c05846 (PMC11261559; doi:10.1021/acsami.4c05846)
Supplement: Supplementary file 1 — am4c05846_si_001.pdf [file am4c05846_si_001.pdf]

Supporting Information for:

Broadening the Scope of Sapofection: Cationic Peptide-Saponin Conjugates Improve Gene Delivery *in vitro* and *in vivo*

Meike Kolster,[a] *Alexander Sonntag,*[a] *Christoph Weise,*[b] *Juan Correa,*[c] *Hendrik Fuchs,*[d] *Wolfgang Walther,*[e] *Eduardo Fernandez-Megia,*[c,*] *and Alexander Weng*[a,*]

[a] Institut für Pharmazie, Freie Universität Berlin, Königin-Luise-Straße 2-4, 14195 Berlin, Germany

[b] Institut für Chemie und Biochemie, Freie Universität Berlin, Thielallee 63, 14195 Berlin, Germany

[c] Centro Singular de Investigación en Química Biolóxica e Materiais Moleculares (CIQUS), Departamento de Química Orgánica, Universidade de Santiago de Compostela, Jenaro de la Fuente s/n, 15782 Santiago de Compostela, Spain

[d] Institut für Laboratoriumsmedizin, Klinische Chemie und Pathobiochemie, Charité – Universitätsmedizin Berlin, corporate member of Freie Universität Berlin and Humboldt-Universität zu Berlin, Augustenburger Platz 1, 13353 Berlin, Germany

[e] Experimental Pharmacology & Oncology Berlin-Buch GmbH, Robert-Rössle-Str. 10, 13125 Berlin, Germany

ef.megia@usc.es (Eduardo Fernandez-Megia)

alexander.weng@fu-berlin.de (Alexander Weng)

Table of Contents

1. Stability Analysis of SO1861-EMCH S3

2. Characterization of Equipped Peptide Scaffolds S5

3. Size and Charge of Nanoplexes S8

4. Electron Microscopy S9

5. Cell Culture S10

6. Flow Cytometry S11

7. Cell Viability S11

8. Statistical Analysis S13

9. References S13

1. Stability Analysis of SO1861-EMCH

The stability of SO1861-EMCH was investigated in different buffers and pH values using ^{1}H nuclear magnetic resonance spectroscopy (^{1}H NMR) (Table S1 and Figure S1). 0.8 to 1.0 mg of SO1861-EMCH was dissolved in 1.2 mL of phosphate-buffered saline (PBS) pH 7.4, 20 mM phosphate buffer (PB) pH 7.4, 20 mM PB pH 7.0, H_2O, 20 mM citrate pH 6.0, or 20 mM citrate pH 4.5 and stirred at 25 or 37 ºC. Aliquots (0.40 mL) were collected at 2, 6, and 24 h, lyophilized, and analyzed by ^{1}H NMR (500 MHz, CD_3OD). The stability/degradation of the hydrazone and maleimide groups was determined by relative integration of the signal at 7.70-7.60 ppm (hydrazone) and 6.82 ppm (maleimide).

^{1}H NMR experiments were recorded on a 11.7 T Bruker DRX 500 spectrometer, acquiring 64 scans, with a pre-scan delay (d1) of 1.6 s, and an acquisition time (aq) of 4 s at 300 K. MestReNova 14.2 software (Mestrelab Research) was used for spectra processing.

Table S1. Extent of hydrolysis (%) of hydrazone and maleimide groups in SO1861-EMCH with time at room temperature in different buffers and pH values. Hydrolysis (%) determined by ^{1}H NMR.

**Buffer**<br>**pH**		**PBS**<br>**7.4**	**PB**<br>**7.4**	**PB**<br>**7.0**	**H_2O**	**Citrate**<br>**6.0**	**Citrate**<br>**4.5**
	2 h	0	0	0	0	0	3
**Hydrazone**	6 h	0	0	0	0	0	6
	24 h	0	0	0	0	0	29
	2 h	0	37	0	0	0	0
**Maleimide**	6 h	0	40	11	6	0	0
	24 h	0	42	54	47	0	11

PBS: phosphate buffered saline, PB: 20 mM phosphate buffer, Citrate: 20 mM citrate buffer.

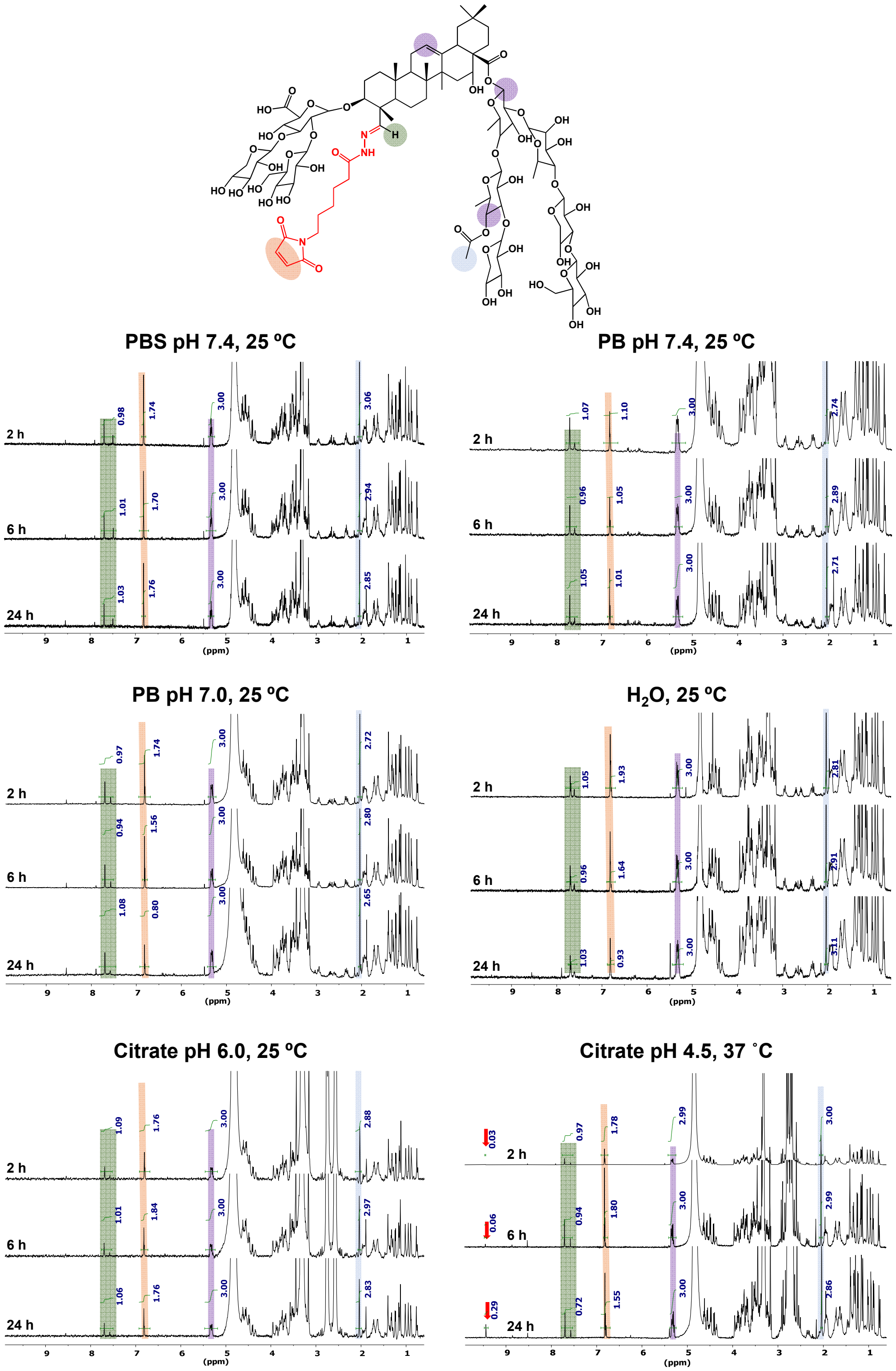

Figure S1. ^{1}H NMR (500 MHz, CD_3OD) spectra of SO1861-EMCH dissolved in different aq media (PBS pH 7.4, 20 mM PB pH 7.4, 20 mM PB pH 7.0, H_2O, 20 mM citrate pH 6.0, 20 mM citrate pH 4.5). After 2, 6, or 24 h at room temperature or 37 ºC, samples were lyophilized, and analyzed by ^{1}H NMR.

2. Characterization of Equipped Peptide Scaffolds

Equipped peptides K_{16}Ceq0.25, K_{16}Ceq0.5, K_{16}CPEGeq0.25, and K_{16}CPEGeq0.5 were characterized using matrix-assisted laser desorption/ionization (MALDI) time-of-flight (TOF) mass spectrometry (MS) (Figure S2). An UltrafleXtreme instrument (Bruker Daltonics, Germany) was used in positive ion-mode. α-Cyano-4-hydroxycinnamic acid (HCCA, Thermo Fisher Scientific™, USA) was used as matrix, mixed 1:1 with the samples and deposited using the dried-droplet-method. Measurements were done in linear mode and mass-to-charge-ratios (m/z) are reported as M+H (average) values.

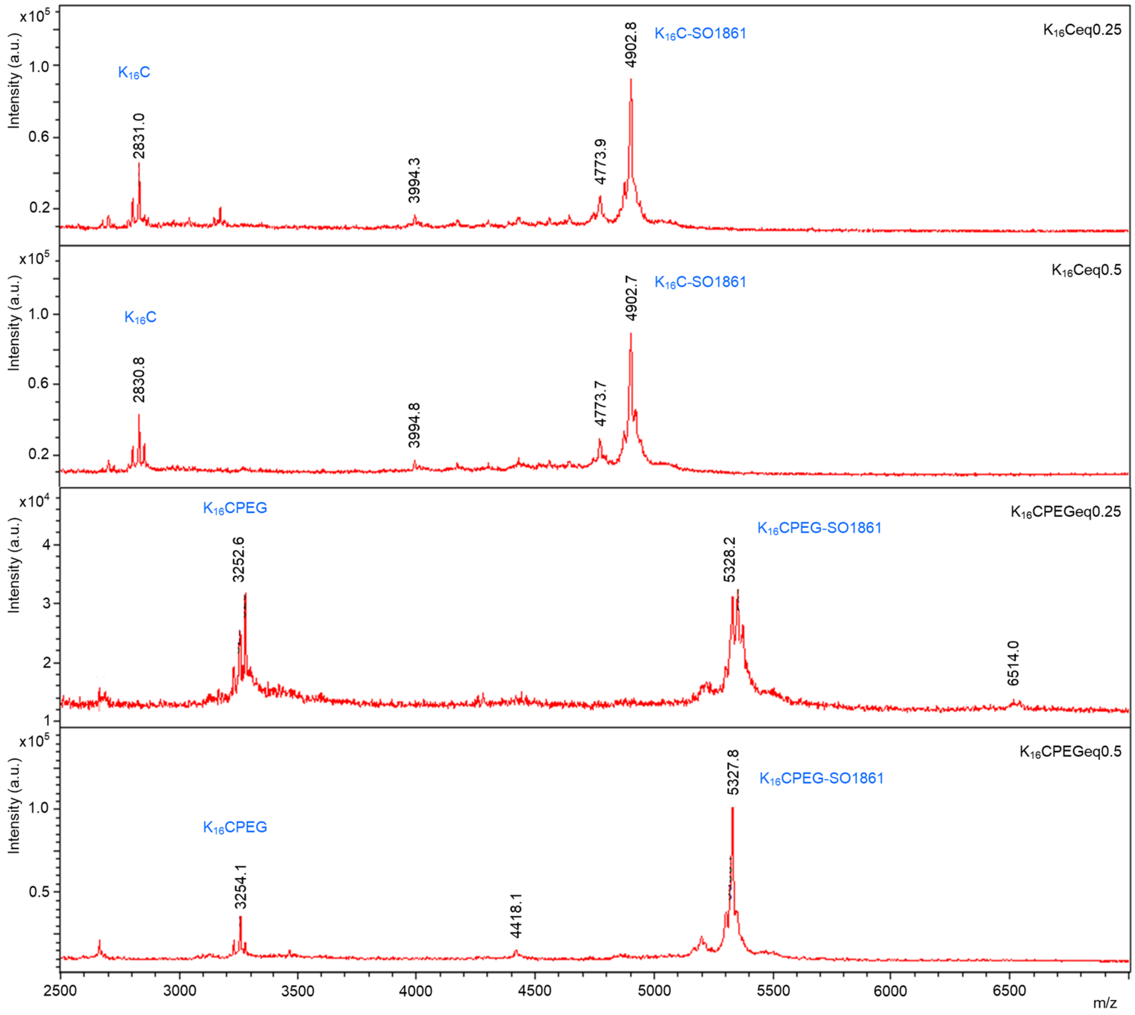

Figure S2. MALDI-MS spectra of SO1861-equipped peptide scaffolds K_{16}Ceq0.25, K_{16}Ceq0.5, K_{16}CPEGeq0.25, and K_{16}CPEGeq0.5 (from top to bottom). Conjugation of SO1861-EMCH to all peptide scaffolds was confirmed (conjugate peak m/z ≈ peptide peak *m/z* +2071). Peaks with $\Delta m/z = -129$ relative to the conjugate peaks represent peptides which lost one lysine. Peaks with $\Delta m/z \approx -909$ relative to the conjugate peaks represent peptide-SO1861 conjugates which lost the sugar chain at C-28.

An LC-MS-Triple Quadrupole 6400 (Agilent Technologies®, USA) was used to evaluate the presence of unreacted SO1861-EMCH in the reaction mixture (Figure S3). HPLC was performed using a Kinetex, 2.6 µm C18, 100 Å, 100×4.6 mm column (Phenomenex, Germany) and a water, 0.01% formic acid (A)/acetonitrile, 0.01% formic acid (B) gradient from 30% to 50% B over 14 min. Flow rate was 0.7 mL/min and sample injection volume was 5.0 µL. Electrospray ionization (ESI)-MS-detection was done in negative ion mode. MestReNova 14.1 software (Mestrelab Research) was used for spectra processing.

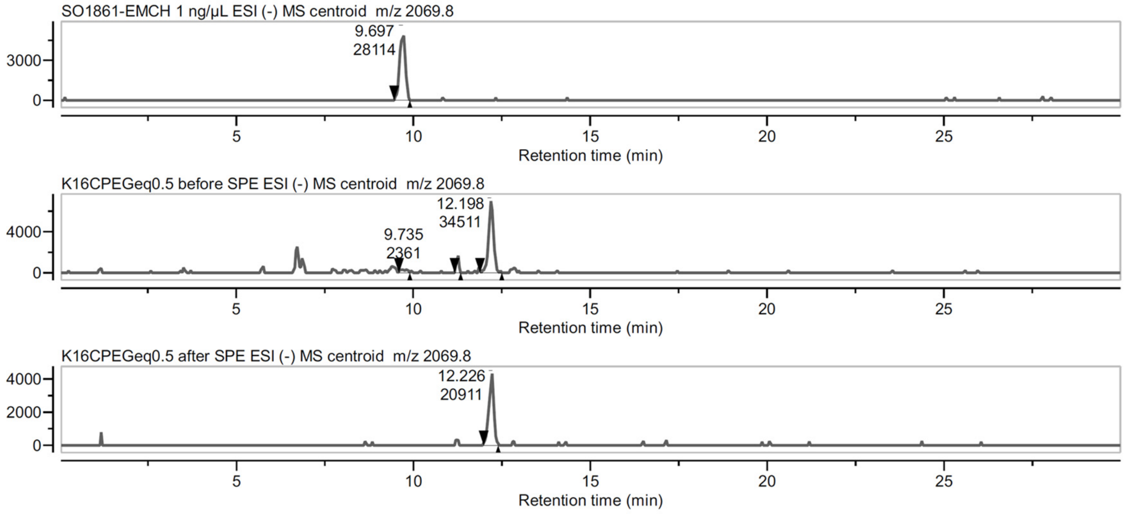

Figure S3. LC-MS-analysis of SO1861-equipped peptide scaffolds. Chromatograms of *m/z* 2069.8 ± 0.25 (representing SO1861-EMCH) in LC-MS analysis. Chromatograms are derived from MS detection data showing the appearance of ions with *m/z* 2069.8 ± 0.25 over the course of the HPLC run. Peaks are labelled with retention time and peak area (bottom number). Panels from top to bottom: SO1861-EMCH 1 ng/µL, K_{16}CPEGeq0.5 before SPE, K_{16}CPEGeq0.5 after SPE. After purification, no free SO1861-EMCH was detected. Peaks at 12.2 min represent SO1861-EMCH which is released from the equipped peptide scaffold during MS-detection.

3. Size and Charge of Nanoplexes

The hydrodynamic diameter (D_h) and zeta(ζ)-potential of all nanoplexes were measured by dynamic light scattering (DLS) and laser Doppler microelectrophoresis using a Malvern Zetasizer Nano ZS instrument (Malvern Instruments, UK), equipped with a 4 mV HeNe laser, 633 nm, at a fixed scattering angle of 173°. For each nanoplex, 2.5 µg DNA were complexed in a total volume of 50 µL as described above. The nanoplex solution was incubated at room temperature for 30 min and transferred into a disposable UV-transparent micro cuvette (SARSTEDT AG & Co. KG, Germany) for size measurements. Cumulants analysis, as defined in ISO 13321:1996 and ISO 22412:2008, was used for the analysis of the autocorrelation functions by the Zetasizer software, producing a mean value for the D_h (Z-Average) and a width parameter of the monomodal curve known as polydispersity index (PdI).[1] For ζ-potential measurements, the nanoplex solution was diluted with the buffer that was used for nanoplex formulation to a final volume of 800 µL before transferring to a folded capillary cell (DTS1070, Malvern Panalytical Ltd, UK). Z-potential values were calculated by the Zetasizer software from the measured electrophoretic mobility using the Henry equation and the Smoluchowski approximation.[1] Each measurement was performed three times with a minimum number of 10 sub-runs per measurement.

Evidence of the stability of the nanoplexes toward FBS in medium was obtained by analyzing the DLS size distribution of the nanoplex K16Ceq0.5 incubated with increasing concentrations of FBS in culture medium. Aliquots of 50% v/v FBS in DMEM were sequentially added to the K16Ceq0.5 nanoplex (pEGFP-N3, N/P 10, 0.42 mg/mL in 10 mM HEPES pH 7.1) in an eppendorf tube. The nanoplex stability was assessed in terms of variations in the DLS size distribution (Figure S4). Complete stability was confirmed

at 2.5% FBS, with a marginal size increase at 5% FBS. Assessment of the stability at higher FBS concentrations is hampered by interference of intense FBS signals.

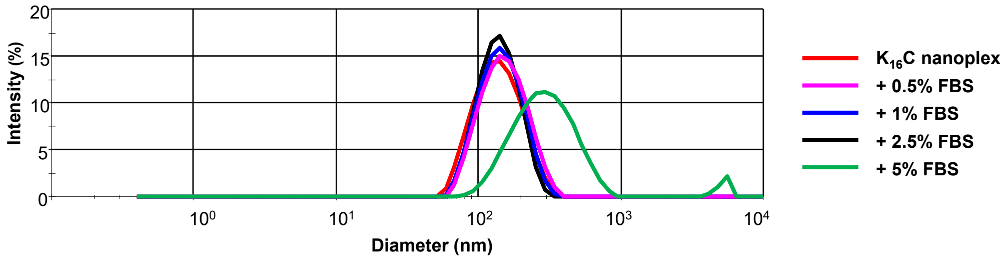

Figure S4. DLS assessment of the stability of K16Ceq0.5 nanoplex (N/P 10, 0.42 mg/mL pEGFP-N3 in 10 mM HEPES pH 7.1) toward FBS in culture medium.

4. Electron Microscopy

For cryogenic transmission electron microscopy (Cryo-TEM), perforated carbon film-coated microscopic 200-mesh grids (either R1/4 or R1.2/1.3 batches of QUANTIFOIL®, MicroTools GmbH, Germany) were cleaned with chloroform and hydrophilized by glow discharge before 4 µL aliquots of the nanoplex solutions (0.3 mg/mL complexed DNA) were applied to the grids. Samples were vitrified by automatic blotting and plunge freezing with a FEI Vitrobot Mark IV (Thermo Fisher Scientific™, USA) using liquid ethane as cryogen. The vitrified specimens were transferred to the autoloader of a Talos Arctica™ transmission electron microscope (Thermo Fisher Scientific™, USA), which is equipped with a X-FEG field emission gun and operated at 200 kVacceleration voltage. Micrographs were acquired on a Falcon 3 direct electron detector (Thermo Fisher Scientific™, USA) using the 100 µm condensor aperture at a nominal magnification of 28000× corresponding to a calibrated pixel size of 3.75 Å per pixel. Nanoplex sizes were measured using ImageJ (version 1.53k, https://imagej.net/).[2]

5. Cell Culture

Cell lines A2058 (ATCC® CRL-11147™), Huh-7 (ATCC® PTA-4583™), ECV-304 (DMSZ ACC 310), HCT 116 (ATCC® CCL-247™), HEK293 FT (Invitrogen® R70007), Hepa 1-6 (ATCC® CRL-1830™), MDA-MB-468 (ATCC® HTB-132™) and Neuro-2a (DMSZ ACC 148) were routinely cultivated at 37 °C in a humidified 5% CO_2 atmosphere. HCT 116 cells were cultivated in Gibco™ McCoy's Modified 5A Medium (Thermo Fisher Scientific™, USA). BioWhittaker® Dulbecco's Modified Eagle Medium (1.0 g/L glucose) (Lonza Group, Switzerland) was used as culture medium for Neuro-2a cells and all other cell lines were kept in BioWhittaker® Dulbecco's Modified Eagle Medium (4.5 g/L glucose) (Lonza Group, Switzerland). All culture media were supplemented with 10% fetal bovine serum (FBS Superior, Bio&SELL GmbH, Germany) and contained 2 mM alanyl-L-glutamine, either directly formulated in the medium or supplemented with UltraGlutamine™ I Supplement (Lonza Group, Switzerland).

6. Flow Cytometry

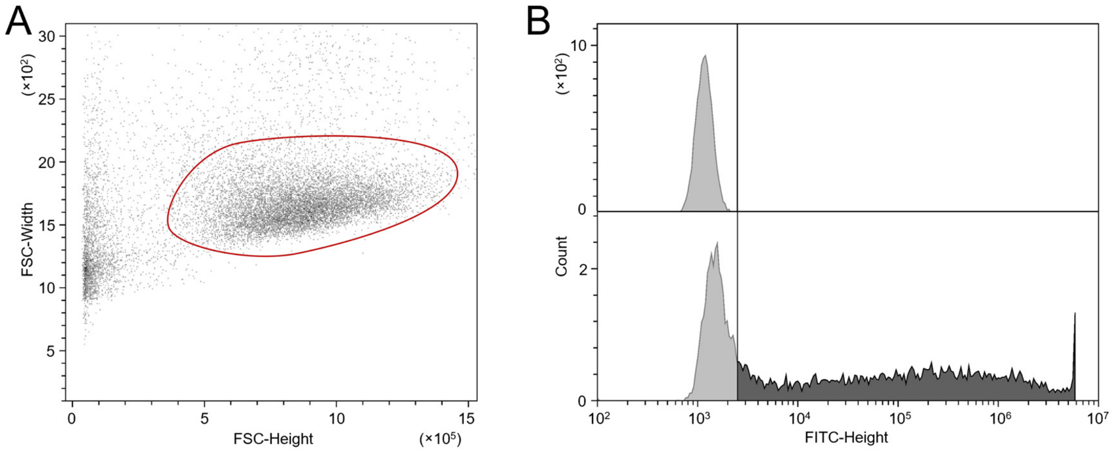

Figure S5. Gating of singlets (single, intact cells), indicated by red circle in the dot plot (A). Evaluation of eGFP-expression. The autofluorescence of a blank (untreated) cell population (upper panel) is used to establish the threshold for eGFP-expression. All cells exhibiting FITC-height values above the threshold were considered transfected as depicted by the dark grey color in the lower panel.

7. Cell Viability

Impedance-based measurements were performed to determine cell viability during transfection. Two E-Plates L8 (ACEA Biosciences, Inc, San Diego, United States) were filled with 150 µL of cell culture medium per well to measure the blank impedance. Afterwards, 20000 cells for cell lines Hepa 1-6 and MDA-MB-468, and 10000 cells for cell lines Neuro-2a, A2058 and HEK293 FT in a volume of 400 µL were added per well. Cells were incubated using the regular cultivation conditions while the impedance (the electric resistance caused by the adhesion of cells on the well surface) was measured every 10 min with the ACEA RTCA iCelligence system (ACEA Biosciences, Inc., San Diego, United States). Data was analyzed by the RCTA data analysis software. After a

24 h incubation period, nanoplex-containing transfection medium (200 ng complexed DNA in 40 µL) and optionally SO1861-EMCH (10 µL, final concentration 2 µg/mL) were added and cell viability was analyzed for another 48 h.

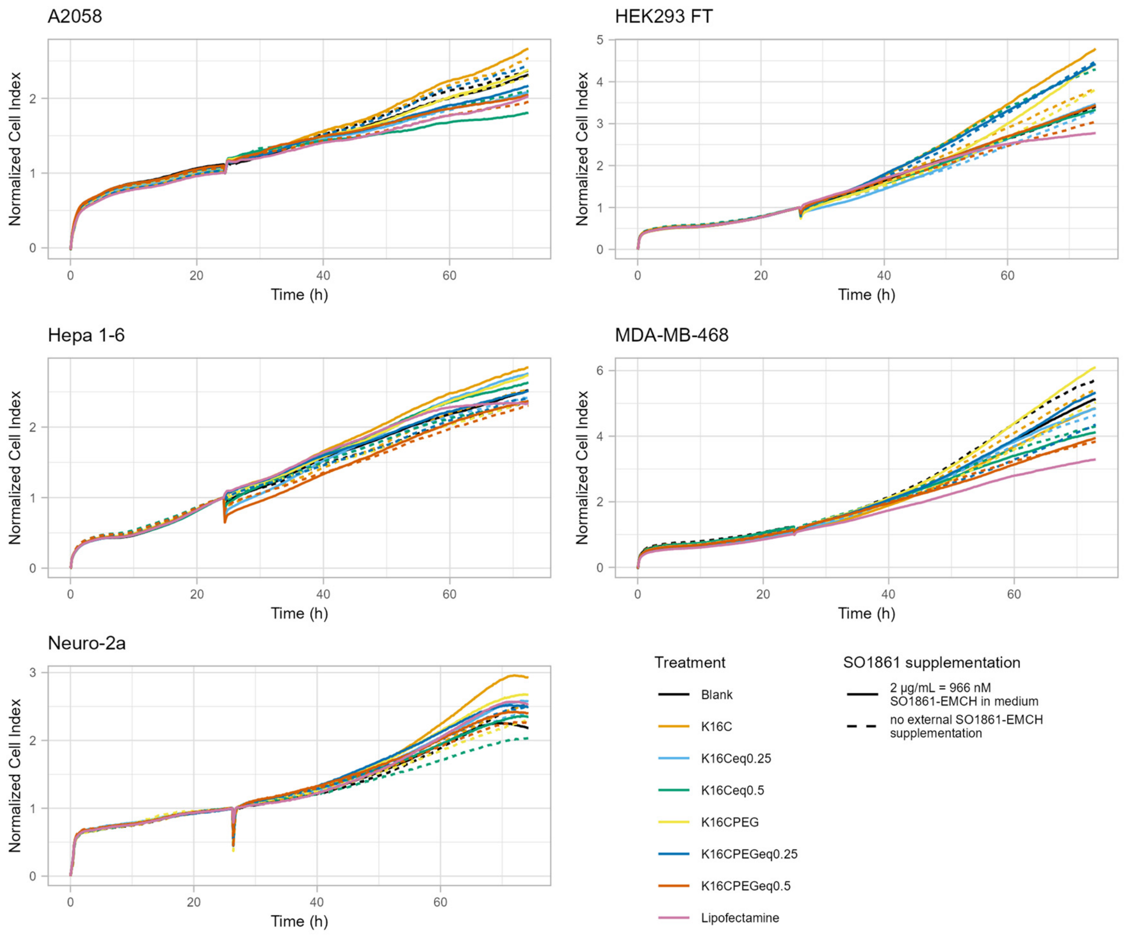

Figure S6. Tolerability *in vitro*. Cell viability indicated by normalized cell index during transfection in various cell lines. Nanoplexes were added 24 h after seeding the cells (evident by the vertical spike in the curves) and cell growth and viability was monitored for the following 48 h. Cell indices were normalized to the cell index at the time of transfection. No distinct toxic effects were observed, but slightly impaired cell growth was detected for Lipofectamine, K_{16}Ceq0.5, and K_{16}CPEGeq0.5 transfections.

8. Statistical Analysis

Statistical analysis was perfomed using R: A Language and Environment for Statistical Computing.[3,4] Normality was assessed with Shapiro-Wilk test assuming a 95% confidence level. If normal distribution was confirmed, statistically significant differences between groups were determined with unpaired, two-sided Student`s *t*-test. If data was not normally distributed, Wilcoxon signed-rank tests were performed to investigate differences. Grubbs's test was used to test for outliers assuming a 95% confidence level.

9. References

1. Malvern Instruments Ltd., Zetasizer Nano User Manual, 2013.

2. Schneider, C. A.; Rasband, W. S.; Eliceiri, K. W. NIH Image to ImageJ: 25 years of image analysis. *Nature Methods* **2012**, *9*, 671-675..

3. R Core Team, R: A Language and Environment for Statistical Computing, R Foundation for Statistical Computing, URL https://www.R-project.org/, 2024.

4. Xu, S.; Chen, M.; Feng, T.; Zhan, L.; Zhou, L.; Yu, G. Use ggbreak to Effectively Utilize Plotting Space to Deal With Large Datasets and Outliers. *Frontiers in Genetics* **2021**, *12*, 774846..
